# Adiabatic Connection Methods Applied to Molecular Crystals

**DOI:** 10.1021/acs.jctc.5c01918

**Published:** 2026-05-05

**Authors:** Eduardo Fabiano, Fulvio Sarcinella, Fabio Della Sala, Chiara Ribaldone, Lorenzo Donà, Bartolomeo Civalleri, Lorenzo Maschio

**Affiliations:** † Institute for Microelectronics and Microsystems (CNR-IMM), Via Monteroni, Campus Unisalento, Lecce 73100, Italy; ‡ Center for Biomolecular Nanotechnologies, Istituto Italiano di Tecnologia, Via Barsanti 14, Arnesano, Lecce 73010, Italy; § Department of Chemistry and NIS Center, University of Torino, Via P. Giuria 5, Torino 10125, Italy

## Abstract

We extend adiabatic connection models (ACMs) derived from the Møller–Plesset adiabatic connection (MPAC) formalism, previously applied only to finite systems, to periodic molecular crystals. Lattice energies for 19 representative systems are computed and compared with periodic MP2, high-level reference, and experimental data. The tested ACMs achieve accuracies comparable to state-of-the-art dispersion-corrected hybrid density functionals and come close to those of correlated wave function methods. Among them, the HFAC24 model, which is a post-Hartree–Fock parameter-free correlation expression that correctly recovers both the uniform electron gas and strong-interaction limits, is the only one with accurate binding energies and accurate total energies. The results in this work demonstrate that MPAC-based ACMs provide an accurate and transferable framework for modeling molecular-crystal energetics and represent a robust, systematically improvable route for developing correlation models for extended systems.

## Graphical abstract

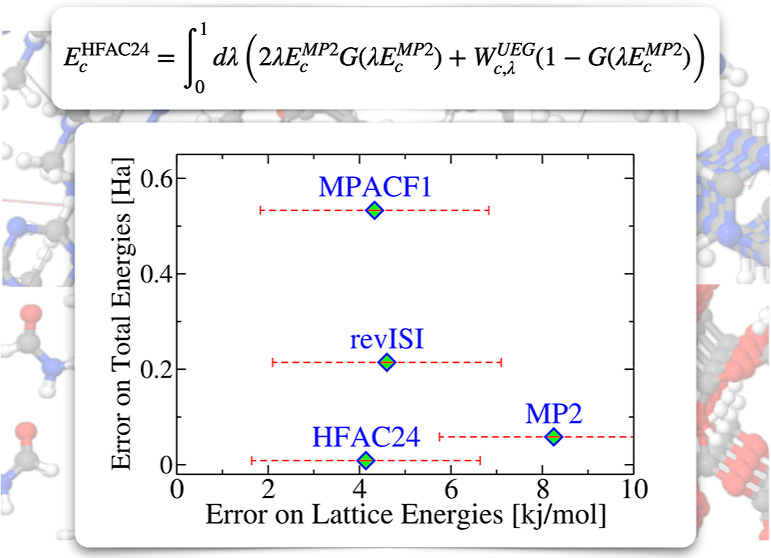

## Introduction

Molecular crystals play a central role in chemistry, materials science, and pharmaceutical development owing to their characteristic optical, electronic, and mechanical properties.^1–6^ This behavior is determined by a subtle balance of noncovalent interactions that control molecular packing.^7–10^ Polymorphism, in addition, can profoundly influence solubility, bioavailability, and charge-transport properties by altering these packing motifs.^10–13^

First-principles characterization and prediction of crystal structures are therefore fundamental for understanding molecular solids and for computational materials discovery.^14–21^ Reliable predictions require accurate lattice energies, since the relative stabilities of polymorphs often differ by only a few kJ/mol.^22^ These small energy separations make lattice-energy calculations a difficult task for electronic-structure theory,^23^ especially because several contributions and assumptions enter in their evaluation, such as thermodynamic effects, entropy contributions, and nuclear quantum effects. However, the most relevant contribution is the electronic lattice energy which requires a careful and balanced treatment. In fact, achieving precision is challenging because cohesion in molecular crystals may arise from multiple noncovalent interactions: electrostatic, induction, hydrogen-bonding, and dispersive forces. A reliable theoretical approach must treat these forces on an equal footing.

In practice, dispersion-corrected density functional theory (DFT) remains the most widely employed approach for these systems, offering a favorable compromise between cost and accuracy.^10,24–27^ Nevertheless, the performance of DFT depends critically on the chosen exchange–correlation functional and dispersion model,^26,28,29^ and even closely related variants may produce qualitatively different energetic orderings or deviate from experiment. Although modern hybrid and double-hybrid functionals have improved the situation,^30–35^ the lack of systematic error control continues to limit predictive power.

To overcome these limitations, correlated wave function methods have been increasingly explored. High-accuracy benchmark lattice energies have been obtained from diffusion Monte Carlo (DMC)^23,36^ and coupled-cluster theory with single, double, and perturbative triple excitations [CCSD­(T)].^37–39^ Computationally cheaper alternatives such as the random phase approximation (RPA)^40–42^ and second-order Møller–Plesset perturbation theory (MP2)^43–45^ have been successfully applied to molecular crystals. In particular, MP2, with its $O\left(N^{5}\right)$ scaling, is computationally efficient and generally reliable for electrostatically dominated systems. Yet, it tends to overestimate dispersion interactions in π-stacked or highly polarizable systems and deteriorates for extended electronic structures.^46,47^

Several regularization schemes have been proposed to mitigate these deficiencies.^48–51^ Among them, the Møller–Plesset adiabatic connection (MPAC) framework offers a theoretically rigorous and computationally efficient route to improved correlation energies.^52,53^ MPAC theory constructs the correlation energy as an integral over the coupling constant connecting the Hartree–Fock (HF) and fully interacting systems. By interpolating between the weak- and strong-coupling limits, the resulting adiabatic connection models (ACMs) incorporate beyond-MP2 curvature effects directly at the wave function level while retaining the favorable scaling of MP2.^53^ These functionals provide correlation energies that naturally account for dispersion without empirical corrections and have demonstrated near-chemical accuracy for a broad range of noncovalent interactions.^53–57^ To date, however, ACMs have been applied and tested only for finite molecular systems.

In this work, we extend the application of MPAC models to periodic molecular crystals and assess their performance for lattice-energy prediction. Several ACM variants are benchmarked against MP2 and high-level reference data from the X23 data set.^58^ We find that standard MP2 systematically overbinds molecular crystals, whereas most ACMs overcorrect this tendency, leading to slight underbinding. However, models such as HFAC24,^54^ constructed to properly recover both the uniform-electron-gas (UEG) and the strong-correlation limits, can yield a balanced and transferable description across all systems. These results demonstrate that MPAC-based methods can provide an efficient and systematically improvable framework for accurate modeling of molecular-crystal energetics.

## Method

In this study, we computed the lattice energies for a representative subset of molecular crystals from the X23 benchmark set^58^ (see below for details). The lattice energy, *E*_latt_, is defined as the sum of the cohesive and molecular relaxation energies

1 $$E_{latt}=E_{coh}+E_{relax}$$

where

2 $$E_{coh}=E_{cell}/N-E_{mol,ghosts}^{\left[crystal\right]},,E_{relax}=E_{mol}^{\left[crystal\right]}-E_{mol}^{\left[gas\right]}$$

In eq 2 *E*_cell_ is the total energy per unit cell from the periodic calculation, *N* is the number of molecules per cell, *E*_mol,ghosts_^[crystal]^ is the energy of an isolated molecule constrained to the crystal geometry surrounded by ghosts atoms up to a distance of 4 Å, *E*_mol_^[crystal]^ is the energy of an isolated molecule constrained to the crystal geometry, and *E*_mol_^[gas]^ is the energy of the fully relaxed gas-phase monomer. All geometries were taken from B3LYP-D*/TZVP optimizations,^59–61^ which yield improved crystal structures^62^ relative to the PBE-D geometries used in the original X23 compilation.^58^ For instance, when comparing the predicted unit cell volumes with respect to the recently back-corrected experimental data of the X23b data set^63^ B3LYP-D* gives a MARE % of 1.9%, while for the PBE0-MBD and PBE-MBD methods as reported in ref 30 the MARE % is 2.4% and 5.9%, respectively. The adopted crystalline and molecular structures are reported in Section S6.

Lattice energies were evaluated at the MP2 level, using the periodic local-MP2 approach^64,65^ (see below for details), and with several ACMs: ISI,^66,67^ revISI,^68^ MPACF1,^69^ and HFAC24.^54^ The SPL2 model^69^ was also investigated. However, we found that it does not satisfy the size-extensivity property (see Section S5) and it is therefore not applicable to periodic calculations, as results depend on the cell folding.

All the ACM methods include the exchange contribution exactly, whereas the correlation energy is expressed as a nonlinear interpolation of the adiabatic connection curve

3 $$E_{c}=F\left(E_{x},E_{c}^{MP2},\mathrm{W̲}\left[n\right]\right)$$

where F is a model-dependent nonlinear function, *E*_*x*_ is the exact exchange energy, *E*_c_^MP2^ is the MP2 energy, and W̲ ­[*n*] is a set of functionals of the density *n* that describe the strong-interaction limit. The latter are

4 $${\underset{\text{\_}}{W}}^{MPAC}\left[n\right]=\left\{W_{c,\infty }^{HF}\left[n\right],W_{1/2}^{HF}\left[n\right],W_{3/4}^{HF}\left[n\right]\right\}$$

where

5 $$W_{c,\infty }^{HF}\left[n\right]=E_{el}\left[n\right]+E_{x}$$

in the case of MPAC models (i.e., MPACF1 and HFAC24) or

6 $${\underset{\text{\_}}{W}}^{DFTAC}\left[n\right]=\left\{W_{\infty }\left[n\right],W_{\infty }^{\prime }\left[n\right]\right\}$$

in the case of DFT adiabatic connection (DFTAC) models (i.e., ISI and revISI). The definition of all these strong-interaction density functionals can be found in literature.^53,54,66,68,70,71^ The functionals *W*_∞_[*n*], *W*_∞_^′^[*n*], *E*_el_[*n*], and *W*_1/2_[*n*] are simple semilocal DFT functionals, which can be evaluated on the HF density as a postprocessing. In particular for the HFAC24 we have (for closed-shell systems)

7 $$E_{el}\left[n\right]=-1.4442\int d^{3}rn{\left(r\right)}^{4/3}F_{el}\left(s\right)$$

8 $$W_{1/2}\left[n\right]=2.8687\int d^{3}rn{\left(r\right)}^{3/2}F_{w}\left(s\right)$$

where in both cases the enhancement factor has the general form^72^

9 $$F\left(s\right)=1+\kappa \left(s\right)-\frac{\kappa \left(s\right)}{1+\mu s^{2}/\kappa \left(s\right)}$$

with κ­(*s*) = *c*/(1 + *s* ^2^) and where μ = 0.399, *c* = 20 for *E*_el_, while μ = 1.601, and *c* = 14 for *W*_1/2_; here $s=\left|\nabla n\right|/\left[2{\left(3\pi^{2}\right)}^{1/3}n^{4/3}\right]$ is the reduced density gradient. For a detailed derivation of these functionals, see ref 54. The functional *W*_3/4_^HF^[*n*], instead, depends on the electronic spin-density at the atomic position,^54^ which is mostly relevant for large atoms.

Note that only HFAC24 depends on all the three ingredients in eq 4, whereas MPACF1 depends only on the first one. In particular the HFAC24 correlation energy can be expressed as^54^

10 $$E_{c}^{HFAC24}=\int_{0}^{1}d\lambda (2\lambda E_{c}^{MP2}G\left(\lambda E_{c}^{MP2}\right)+W_{c,\lambda }^{uegIHF}\left(1-G\left(\lambda E_{c}^{MP2}\right)\right)$$

In eq 10 the correlation integrand is an interpolation between the weak-interaction limit (i.e., 2λE_c_^MP2^) and *W*_c,λ_^uegIHF^ which depends on all the three functionals in eq 4 and describes the UEG behavior; the function 0 ≤ *G*(λ*E*_c_^MP2^) ≤ 1 is an interpolation function.^54^ In this way the HFAC24 functional can describe both molecules, strongly correlated systems and metals.^54^

In this work, all ACM calculations were performed in the MPAC framework, i.e. nonself-consistently on HF orbitals and densities.

Functionals developed in the MPAC context are pure post-HF methods. Note that the ISI and revISI models had instead been originally developed in a different framework, namely the DFTAC. Nevertheless, they have already often been used with HF orbitals, showing reasonably good performance.^57,73,74^ Thus, we will consider them in our MPAC assessment. Other ACMs specifically developed for the DFTAC framework, such as the recently developed genISI2 functional,^70^ have instead not been considered here, because they explicitly require DFT orbitals and eigenvalues. Note, also, that for all investigated molecular crystals the size-consistent correction (SCC)^74^ is zero, as there is just one type of molecule in the unit cell.

Functionals based on the MPAC are constructed in order to satisfy two important limits: the MP2 slope at weak coupling and the strictly correlated electron regime at large coupling. Dispersion forces, investigated in this work, are in between these two limits, and thus the overall accuracy of the MPAC depends on the interpolation formula, e.g. eq 10 in the manuscript. The UEG limit, fundamental for the description of the correlation energy of large systems, enters in the HFAC24 correlation functional in a complex and nonlinear mixing with the MP2 correlation, so that the HFAC24 total correlation energies are usually larger (in absolute value) than the MP2 ones, whereas interaction energies are smaller.^54^ Note that ISI, revISI and MPACF1 fail to reproduce the UEG limit, which is instead recovered by HFAC24.^54^

In the case of cohesive energy calculations, which involve periodic simulations as well as molecular calculations with ghost atoms, to obtain accurate MP2 energies at an affordable computational cost, we performed a complete basis set (CBS) limit extrapolation, starting from (p-aug-)­cc-pVDZ^75^ and (p-aug-)­cc-pVTZ^75^ results (see Computational Details below). Additionally, to ensure a balanced treatment of all energy components, which is especially important in the computation of the ACMs, we have extrapolated all other energies in a similar way as the MP2 one. Instead, relaxation energies were computed with the cc-pV5Z^75^ basis set (which can be considered converged also without extrapolation) for every method (see Table S1). As the periodic local MP2 calculations are performed in real space from localization of the periodic wave function, other extrapolations (e.g., *k*-point extrapolations to the periodic limit) are not required, and the direct space thresholds and screenings ensure convergence of direct space contributions.^76^

The adopted computational procedure proved to be efficient to yield MP2 lattice energies of consistently good quality. This is illustrated in Figure 1, where our CBS-extrapolated MP2 results are compared with those reported in refs 38 and 49 . Despite some minor differences, probably ascribable to the different underlying crystal geometries and to the employed basis set (ref 38 uses an extrapolation from aug-cc-pVTZ and aug-cc-pVQZ, ref 49 uses an extrapolation from cc-pVTZ and cc-pVQZ with frozen core), a good agreement is found across the entire test set. The figure also displays the evolution of MP2 errors as a function of the basis set. This highlights that the apparent good MP2 performance observed when using the cc-pVTZ basis is deceptive: the smaller deviations from experiment mainly arise from a fortuitous cancellation of errors due to the systematic underestimation of MP2 lattice energies at this level, as confirmed by their trend relative to the DZ data. This behavior emphasizes the necessity of proper extrapolation toward the complete CBS limit to achieve physically meaningful and quantitatively reliable results.^38,49^

**Figure 1:**
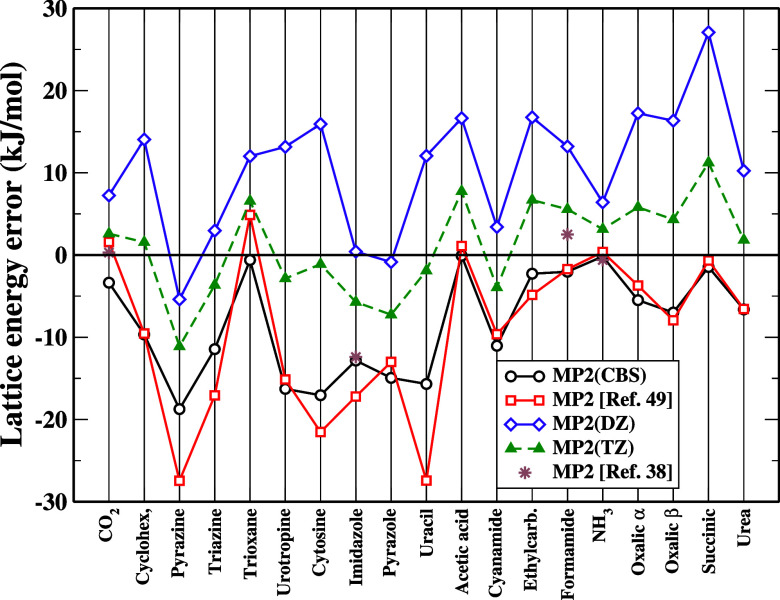
Deviation from experiment (in kJ/mol) of MP2 energies obtained in refs 38 and 49 and in this work with different basis sets as well the CBS extrapolation. Note that the results of ref 49 have been obtained using different geometries than in the present study. The MP2 value of Pyrazole for ref 49 has been corrected according to revised data.^77^

### Test Set

The test set considered in this work comprises 19 molecular crystals selected from the X23 benchmark database.^58^ The chosen subset includes CO_2_, 1,4-cyclohexanedione, pyrazine, triazine, trioxane, urotropine, cytosine, imidazole, pyrazole, uracil, acetic acid, cyanamide, ethylcarbamate, formamide, NH_3_, oxalic acid α, oxalic acid β, succinic acid, and urea.

This set provides a compact yet chemically diverse representation of the X23 database, encompassing crystals characterized by qualitatively different types of noncovalent interactions.^62^ In particular, it spans systems dominated by dispersion forces (CO_2_, 1,4-cyclohexanedione, pyrazine, triazine, trioxane, urotropine), mixed hydrogen-bonding and dispersion (cytosine, imidazole, pyrazole, uracil), and hydrogen-bonded networks (acetic acid, cyanamide, ethylcarbamate, formamide, NH_3_, oxalic acid α, oxalic acid β, succinic acid, urea). The inclusion of multiple bonding motifs, spanning from H-bond to π-stacked heteroaromatics, ensures a balanced assessment of exchange–correlation effects across interaction regimes. A few systems from the original X23 set, namely benzene, naphthalene, anthracene, and adamantane, were notably excluded. Due to technical limitations, it was not possible to accurately localize the solution in the large cc-pVTZ basis set using the CRYSTAL23 code.

### Periodic Local MP2

The MP2 correlation energy was computed using a periodic local MP2 (LMP2) approach, formally analogous to local correlation methods developed for finite systems.^78,79^ In this framework, the occupied and virtual subspaces of the HF reference are represented by localized Wannier functions, which enable the spatial decay of correlation amplitudes to be exploited. The locality of these functions, together with the use of translational symmetry, permits systematic truncation of pair domains and summations in the MP2 energy expression, thereby achieving an efficient evaluation of the second-order correlation energy under periodic boundary conditions.

All the periodic Local MP2 calculations in this study were carried out with the CRYSCOR program,^64,65^ which employs density fitting techniques for the fast computation of electron-repulsion integrals.^80^ The virtual space was constructed using a modified orbital-specific virtuals (OSV) algorithm,^76^ in which the canonical virtual manifold is locally optimized and augmented with a small set of diffuse projected atomic orbitals to improve the description of long-range correlation effects.^81^

### Extrapolation of Energy Components

The total energies considered in this work depend on four fundamental quantities: the HF energy *E*_HF_, the exact exchange energy *E*_*x*_, the MP2 correlation *E*_c_^MP2^, and the strong-coupling ingredients of the adiabatic connection, W̲ , from eqs 4 or 6. Among these, *E*_c_^MP2^ exhibits a particularly strong dependence on the basis set size and must therefore be accurately converged toward the CBS limit. The remaining quantities show a comparatively weaker basis-set dependence, but they enter the ACM total energy through nonlinear combinations with the MP2 correlation. Consequently, an unbalanced convergence of individual components may introduce numerical artifacts and compromise the internal consistency of the adiabatic-connection interpolation.

In periodic calculations, however, the computational cost of large basis sets quickly becomes prohibitive. In practice, our calculations could be performed only up to the cc-pVTZ level. To mitigate basis-set incompleteness while preserving accuracy, all quantities entering the ACM functionals were therefore extrapolated to the CBS limit using the cc-pVDZ and cc-pVTZ results. The extrapolation was performed systematically for all energy components according to the two-point power-law expression

11 $$E_{ext}=\frac{E_{TZ}3^{\alpha }-E_{DZ}2^{\alpha }}{3^{\alpha }-2^{\alpha }}$$

where *E*_DZ_ and *E*_TZ_ are the double- and triple-ζ results, respectively, and α is an exponent controlling the rate of convergence toward the CBS limit. An optimized value of α was determined individually for each system and each energy component following the procedure below:

1. For every molecular crystal, we evaluated the molecular-in-crystal energy *E*_mol,ghosts_^[crystal]^ and related ingredients using cc-pV*n*Z basis sets with *n* = D, T, Q, 5. This setup effectively accounts for basis set superposition effects in extended systems. The most expensive part is the HF SCF calculation with *n* = 5: for the largest system considered, it took about 9 days on a single core of a last generation CPU.
2. For the HF and exchange energies as well as for the density-based quantities (the W̲ components), the 5Z values were taken as reference. For the MP2 correlation energies, CBS reference values were obtained according to the extrapolation scheme of ref 82.
3. For each molecule and each energy term, the parameter α was optimized such that eq 11 exactly reproduced the corresponding reference value, as described in point 2 above. The resulting α parameters are summarized in Table S2.

For each energy component, we also computed the average value of α, which can be used in practical applications. This allows to perform quite accurate calculations using only DZ and TZ results and may be of help also in cases where energy differences between two distinct systems are of interest (e.g., in polymorph analysis). The values are reported in Table S2.

### Computational Details

HF calculations and corresponding Wannier functions localization were performed using the CRYSTAL23 program package,^83^ which employs atom-centered Gaussian basis functions and can manage both molecular (nonperiodic) and periodic boundary conditions simulations.^21^ All CRYSTAL calculations employed integral accuracy thresholds (TOLINTEG) of 8 8 8 20 60, and the correlation-consistent cc-pVDZ and cc-pVTZ basis sets.^75^ We use a shrinking factor of 6 for both the Monkhorst-Pack and the Gilat nets: this corresponds to 64 *k*-points, which can be considered fully converged and thus no *k*-point extrapolation is required.

The evaluation of the strong-correlation ingredients required for the adiabatic-connection models was carried out using a development version of the code, which includes an implementation for computing the relevant quantities directly from the electron density:

For HFAC24, *W*_c,∞_^HF^[*n*], *W*_1/2_^HF^[*n*], and *W*_3/4_^HF^[*n*] as defined in eqs 9–11 of ref 54; for other ACMs the PC-model-based expressions.^66^

Post-HF calculations at the MP2 level were conducted with the CRYSCOR code, as described above, using a dual-basis scheme.^84,85^ In this approach, the HF reference is obtained with a smaller basis set (here cc-pVDZ or cc-pVTZ), while additional diffuse functions are introduced only during the correlation step. This strategy allows the inclusion of diffuse contributions essential for dispersion interactions while avoiding the convergence issues that frequently occur in self-consistent-field (SCF) calculations with diffuse s- and p-type functions. Because the dual-basis procedure formally violates the Brillouin theorem, a small correction arising from single excitations (the so-called MP1 term) was included via a first-order perturbative estimate.^86,87^ To implement this scheme, the standard cc-pVDZ and cc-pVTZ basis sets were augmented with the diffuse functions of the aug-cc-pV*n*Z family,^88^ with all s-type functions removed and p-type diffuse functions excluded for C, N, and O atoms: the resulting basis sets are then named p-aug-cc-pVTZ and p-aug-cc-pVDZ. This modified basis has been successfully employed in previous studies on molecular crystals, where it was shown to provide a balanced and numerically stable description of long-range correlation effects.^62,89,90^ The computational cost of the LMP2 calculations in the p-aug-cc-pVTZ basis ranges from a few hours, for smaller systems, to ≈20 days, running on a single core.

The calculations used to compute the CBS extrapolation coefficients, the relaxation energies and the reference CCSD(T) used in Figure 3 were carried out using the TURBOMOLE program package.^91^

**Figure 2:**
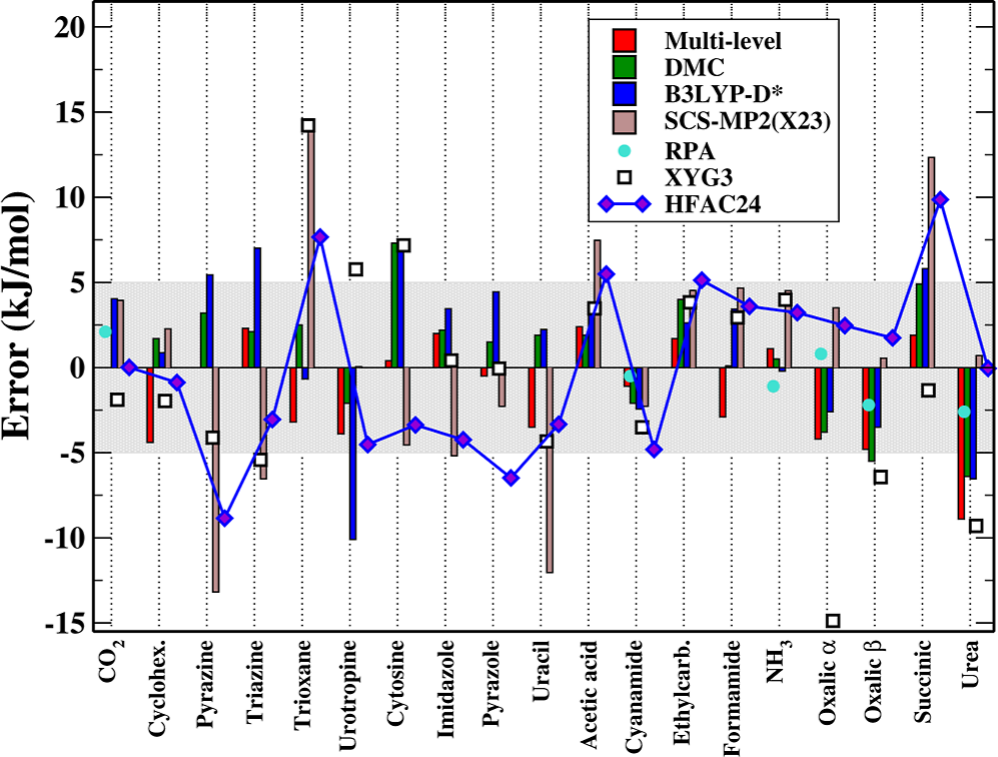
Lattice energy errors with respect to the experimental ref 63 for different methods: multilevel CCSD­(T),^37^ Diffusion Monte Carlo (DMC),^23^ RPA,^40^ XYG3,^93^ B3LYP-D*,^62^ SCS-MP2 with optimized parameters for X23 (SCS-MP2­(X23)),^49^ and HFAC24. The shaded area indicates the expected accuracy of the experimental data (±5 kJ/mol).^63^ SCS-MP2­(X23) value for Pyrazole has been corrected according to revised data.^77^

**Figure 3:**
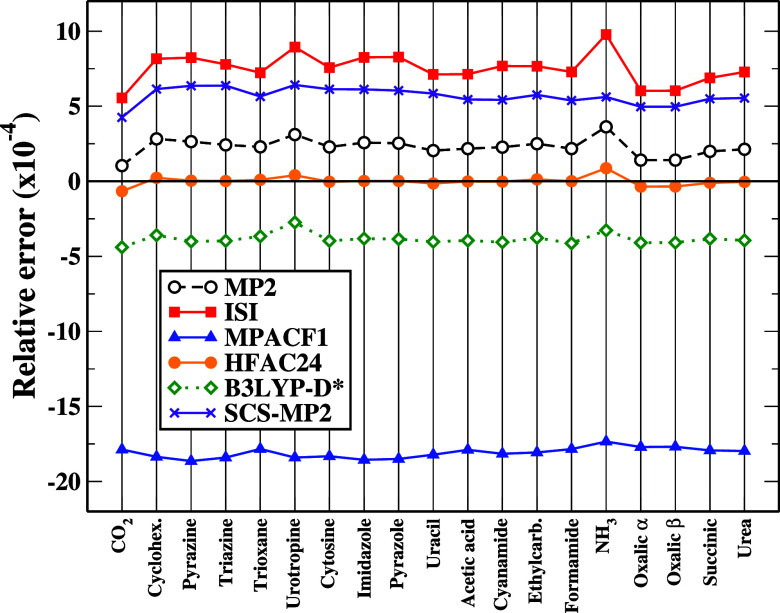
Relative total energy deviation with respect to CCSD­(T), computed for isolated (unrelaxed) molecules with different methods. RevISI results are not reported since, on the scale of the graph, they overlap with the ISI ones. All energies have been computed using the cc-pV5Z basis set. CCSD­(T) energy estimates have been obtained adding to the MP2 cc-pV5Z energies a δ*E*_c_^CCSDT^ correction calculated with the cc-pVTZ basis set. See Figure S2 for absolute relative error.

Finally, the ACM total energies were evaluated using the acmxc script,^92^ which interfaces with CRYSTAL and CRYSCOR or TURBOMOLE output files to assemble all required quantities and compute the corresponding ACM energies and related adiabatic-connection properties. Note that the computational cost of running the ACM script is negligible with respect to that required for the calculation of the HF and MP2 energies: the calculation of the strong-interaction ingredients has approximately the same computational cost of one SCF step of a semilocal density functional theory calculation.

## Results and Discussion

Table 1 summarizes the deviations of computed lattice energies from experimental reference data^63^ for the set of molecular crystals, together with the corresponding statistical indicators. The systems are grouped according to the dominant interaction type, following the classification of Cutini et al.^62^ See also Table S6 with total lattice energies and Table S7 with errors computed with the averaged exponents for the CBS extrapolation.

**Table 1:**
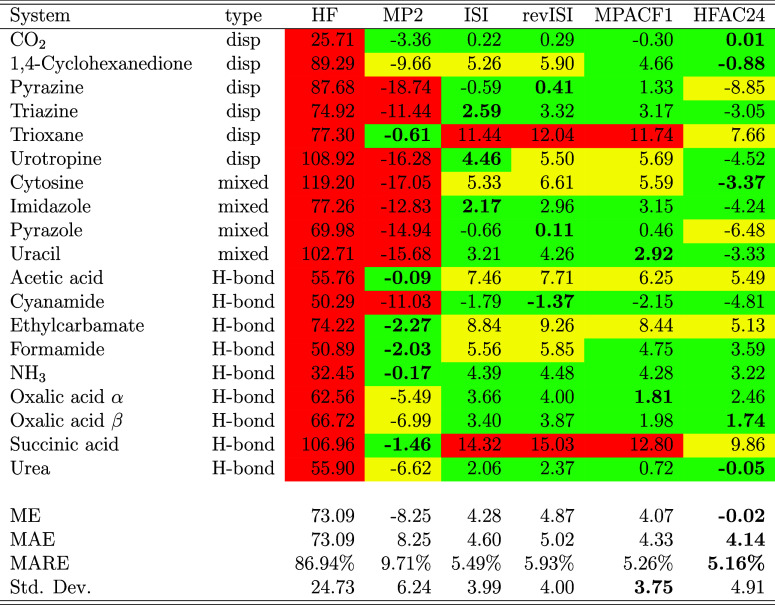
Lattice Energy Errors with Respect to Experimental Reference Data^63^ (in kJ/mol) for All Systems and Methods^a^

a The type of interaction, according to ref 62, is also reported. At the bottom of the table we display the error statistics: mean error (ME), mean absolute error (MAE), mean absolute relative error (MARE), and population standard deviation (std. dev.). Table entries are colored green and yellow if the absolute value of the error is below 5 and 10 kJ/mol, respectively, red otherwise. The smallest error for each system is highlighted in bold style.

As we could expect, due to lack of correlation contributions, the HF approximation largely underbinds all systems, yielding mean absolute errors (MAE) exceeding 70 kJ/mol. Consistent with previous studies on molecular crystals^10,43,49^ the inclusion of dynamical correlation at the MP2 level markedly improves the description, lowering the MAE to 8 kJ/mol. However, MP2 always tends to overestimate the interaction, overbinding in particular dispersion-dominated systems (see Table 2). In fact, while for hydrogen-bonded (H-bond) crystals it yields a mean error (ME) of only 4 kJ/mol, for dispersion and mixed systems it gives MEs of more than 10 kJ/mol, reflecting its well-known tendency to overestimate long-range correlation effects.^10,56^

**Table 2:** Error Statistics (in kJ/mol) with Respect to Experimental Reference Data^63^ for Different Types of Interactions (Defined in Reference 62): Mean Error (ME), Mean Absolute Error (MAE), Mean Absolute Relative Error (MARE), and Population Standard Deviation (Std. Dev.)^a^

system	HF	MP2	ISI	revISI	MPACF1	HFAC24
Dispersion Interaction						
ME	77.30	–10.01	3.90	4.58	4.38	**–1.61**
MAE	77.30	10.01	**4.09**	4.58	4.48	4.16
MARE	115.31%	14.98%	**5.78%**	6.44%	6.38%	6.15%
std. dev.	25.56	6.46	3.97	3.99	**3.84**	5.03
Mixed Interaction						
ME	92.29	–15.12	**2.51**	3.49	3.03	–4.36
MAE	92.29	15.12	**2.84**	3.49	3.03	4.36
MARE	80.65%	13.77%	**2.21%**	2.65%	2.41%	4.36%
std. dev.	19.72	1.53	2.16	2.35	1.82	**1.28**
H-Bond Interaction						
ME	61.75	–4.02	5.32	5.69	4.32	**2.96**
MAE	61.75	**4.02**	5.72	5.99	4.80	4.04
MARE	70.82%	**4.39%**	6.75%	7.04%	5.78%	4.87%
std. dev.	19.45	**3.51**	4.30	4.38	4.21	3.81

a The smallest value for each entry is highlighted in bold style.

The adiabatic-connection-based models (ISI, revISI, MPACF1, and HFAC24) correct the MP2 overbinding to various extents. These models approximately halve the MP2 MAE (4–5 kJ/mol vs 8 kJ/mol), reaching accuracies comparable to the expected experimental uncertainty of lattice energies (5 kJ/mol^10,63^ ). These functionals yield a more balanced performance across interaction types, with most systems within 5 kJ/mol of experiment. Such balanced accuracy indicates that adiabatic-connection-based correlation functionals efficiently capture both dispersion and polarization components of the intermolecular binding, as also observed in gas-phase noncovalent interaction benchmarks.^56^

The HFAC24 model achieves the best overall performance, with an almost vanishing mean error (ME = −0.02 kJ/mol) and the lowest mean absolute relative error (MARE = 5.2%). This is quite relevant considering that HFAC24 is completely nonempirical, whereas and MPACF1 has 2 empirical parameters fitted on dispersion complexes. The superior performance of HFAC24 can be possibly ascribed to its correct recovery, beside the MP2 limit, of both the strong-interaction limit and the UEG behavior, which ensures accurate curvature of the adiabatic-connection integrand and hence a balanced description of different bonding regimes.

A detailed analysis of error decomposition by interaction type (Table 2) further supports these trends. For dispersion systems, the ACM models reduce the MP2 mean error by about an order of magnitude, bringing the MAE down to 4 kJ/mol. For mixed interactions, ISI and revISI achieve MAE below 3 kJ/mol, while HFAC24 attains the smallest standard deviation, reflecting consistent performance across systems. In H-bonded systems, MP2 already performs relatively well, but HFAC24 still provides a slight improvement, reducing the dispersion of errors.

Yet, molecular crystals of large aromatic systems, such as benzene, are not included in the analysis above, as these systems could not be handled by our LMP2 approach with the p-aug-cc-VTZ basis due to technical issues. To provide an estimate of the performance of ACM methods for the benzene crystal, we considered the values reported in ref 49 and the procedure described in Section S3. We found that all ACM functionals correct the MP2 overbinding: HFAC24 improves over 25%, and MPACF1 almost exactly reproduces the experimental results. This is quite a promising and interesting result, as HFAC24 is fully non empirical (and without any dispersion correction), whereas MPACF1 employs two parameters fitted on the dispersion interaction. The present result confirms that MPACF1 can also be applied to molecular crystals and that HFAC24 can be a good starting point for future developments. Adding benzene and similar stacked crystal to Table 2 will not change significantly the discussion, as the MAE of MP2 increases much more than the MAE of ACM methods.

In Figure 2, the lattice energy errors from Table 1 are compared with representative data from the literature. Although this comparison is necessarily qualitative, since the reference data were obtained using different computational protocols, basis sets, and sometimes distinct experimental or optimized geometries, it provides a clear overview of the relative performance of the different methods. The figure confirms that conventional MP2 shows a pronounced overbinding tendency, consistent with its known deficiencies in treating dispersion-dominated interactions. The scaled MP2 variant (SCS-MP2­(X23)),^49^ that was specifically parametrized for the X23 set, while improving somewhat over unscaled MP2 (MAE of 6.5 vs 8.3 kJ/mol), still displays significant scatter and often deviates from experiment beyond the uncertainty range of ±5 kJ/mol. A slightly better behavior is obtained with the double-hybrid XYG3 functional,^93^ which delivers a MAE of 5.0 kJ/mol, but still displays a fairly erratic performance across the various systems.

Adiabatic-connection-based models, particularly HFAC24, instead provide good agreement with the experimental data for most of the systems and achieve accuracies that compare with those of high-level benchmarks such as multilevel CCSD­(T)^37^ and diffusion Monte Carlo (DMC),^23^ which yield MAEs of 2.6 and 2.8 kJ/mol, respectively. Moreover, they display a rather balanced description of hydrogen-bonded, mixed, and dispersion-bound crystals. For the 19 systems under investigation, a similar performance is also obtained by B3LYP‑D*,^62^ that provides a MAE of 4 kJ/mol, and other DFT methods such as PBE0-MBD and PBE-MBD with a MAE of 3.4 and 3.7 kJ/mol, respectively.^30^ Note, however, that these functionals, contrary to HFAC24, contain several empirical parameters.

Overall, these results highlight the robustness of the ACM framework for molecular crystals. All adiabatic-connection functionals deliver consistently accurate lattice energies across the full spectrum of interaction types, achieving accuracy comparable to the best available correlated and quantum Monte Carlo methods. Among them, HFAC24 stands out for exhibiting not only small absolute errors but also an almost vanishing systematic deviation from experiment, indicating a physically balanced treatment of dynamical correlation.

A deeper analysis reveals that HFAC24 attains this level of accuracy with the smallest energy compensation effect among all tested models. This is illustrated in Figure 3, which compares the total molecular energies from the different methods against CCSD­(T) reference data. The correlation energies of most ACM methods as well as MP2 are far from the reference, indicating that the corresponding lattice energy results, which instead display errors one or two orders of magnitude smaller, are strongly influenced by error cancelation. In fact, MPACF1 has been developed and optimized for the calculation of interaction energies so that it cannot be expected to yield accurate total energy values for molecular systems. Similarly, B3LYP-D* and SCS-MP2 have been trained on energy differences and display a non-negligible systematic error for total energies.

On the contrary, only HFAC24 results lie uniformly closer to the reference values indicating that its improved lattice energies do not rely on fortuitous cancelation of errors. This behavior can be directly linked to the theoretical construction of the functional: unlike MPACF1, HFAC24 is fully nonempirical, and unlike (rev)­ISI, it was explicitly formulated within the MPAC framework to recover the correct UEG and strong-interaction limits.

This finding is significant because it demonstrates that HFAC24 achieves high accuracy through a faithful representation of the underlying electronic correlation rather than through empirical parameter tuning or error cancelation. Consequently, it provides a rigorous and transferable foundation for extending adiabatic-connection models to more complex condensed-phase systems, including polymorphic and mixed-interaction molecular crystals.

## Conclusions

Reliable first-principles approaches are essential for understanding the electronic properties of molecular crystals, where subtle energy differences govern key physical and functional properties. However, achieving quantitative accuracy remains challenging, as it requires a balanced description of a broad range of noncovalent interactions.

In this work, we extended the use of adiabatic connection models to extended systems and assessed the performance of several ACMs, grounded in the MPAC framework, for predicting lattice energies of molecular crystals. All of the examined ACMs yield consistent and accurate lattice energies across different interaction types. In particular, the HFAC24 functional exhibits small mean deviations and negligible systematic bias with respect to reference data, indicating a physically balanced description of electron correlation. Preliminary results for large stacked aromatic systems, show that MPACF1 is the most accurate ACM functional, while HFAC24 partially corrects the MP2 overbinding.

Overall, ACMs deliver an accuracy comparable to state-of-the-art dispersion-corrected DFT methods and are not far from that of high-level correlated wave function approaches, with mean absolute errors within the typical experimental uncertainty of lattice energies. Importantly, these methods naturally incorporate dispersion and medium-range correlation, with no need for ad-hoc corrections. Moreover, most approaches are parameter-free, formulated entirely from first-principles, and retain the favorable computational scaling of MP2, making them competitive for periodic systems.

In conclusion, the present findings establish a foundation for the extension of MPAC methods to molecular crystals and other condensed-phase systems. The results demonstrate the robustness of ACMs for solid-state applications and can be used as reference data in future studies. Moreover, given the proven accuracy of ACMs for finite molecular systems, the present assessment of their performance in periodic molecular crystals further supports their future applicability to hybrid environments, such as molecular interfaces and organic–inorganic heterostructures, where a balanced, parameter-free treatment of correlation and dispersion is crucial.

## Supplementary Material



## Notes

Relaxation energies, CBS extrapolation coefficients, result on benzene crystal, additional data on lattice energies, comment on the non size-extensivity of the SPL2 model, crystalline and molecular structures (PDF)
