# Supplementary material for: Adiabatic Connection Methods Applied to Molecular Crystals
Source: J Chem Theory Comput. 2026 May 5;22(10):5164–73. doi: 10.1021/acs.jctc.5c01918 (PMC13217546; doi:10.1021/acs.jctc.5c01918)
Supplement: Supplementary file 1 [file ct5c01918_si_001.pdf]

Supporting information to: Adiabatic Connection Methods Applied to Molecular Crystals

Eduardo Fabiano[1,2], Fulvio Sarcinella[1], Fabio Della Sala[1,2], Chiara Ribaldone[3], Lorenzo Donà[3], Bartolomeo Civalleri[3], Lorenzo Maschio[3]
[1] Institute for Microelectronics and Microsystems (CNR-IMM), Via Monteroni, Campus Unisalento, 73100 Lecce, Italy
[2] Center for Biomolecular Nanotechnologies, Istituto Italiano di Tecnologia, Via Barsanti 14, 73010 Arnesano, LE, Italy
[3] Department of Chemistry and NIS center, University of Torino, Via P. Giuria 5, 10125 Torino, Italy

April 18, 2026

Contents

S1 Relaxation energies **S2**

S2 CBS extrapolation coefficients **S3**

S3 Benzene Crystal **S4**

S4 Additional data on lattice energies **S5**

S5 Non size-extensivity of the SPL2 model **S8**

S6 Geometries **S10**

S1 Relaxation energies

System	HF	MP2	ISI	revISI	MPACF1	HFAC24
CO_2	-1.30	0.11	-0.30	-0.34	-0.67	-0.17
NH_3	0.96	0.73	0.88	0.90	1.27	0.88
Acetic acid	10.94	7.87	8.58	8.64	9.11	8.36
Cyanamide	9.20	6.81	7.16	7.18	7.07	7.03
Ethylcarbamate	7.15	5.78	6.11	6.14	6.39	6.00
Oxalic acid α	30.57	31.48	31.94	32.03	33.61	31.91
Succinic acid	19.64	16.90	17.66	17.74	18.69	17.49
Uracil	17.16	8.00	10.12	10.31	11.20	9.31
1,4-Cyclohexanedione	5.58	0.48	1.39	1.46	0.89	0.79
Formamide	13.41	7.25	8.84	8.99	9.94	8.25
Pyrazine	0.39	0.35	0.37	0.37	0.40	0.36
Triazine	0.90	0.40	0.55	0.56	0.71	0.50
Urea	20.17	16.01	16.88	16.95	17.42	16.61
Cytosine	24.15	16.63	18.37	18.53	19.24	17.70
Imidazole	5.62	3.00	3.64	3.70	4.09	3.41
Oxalic acid β	30.24	34.52	34.08	34.08	35.10	34.34
Pyrazole	5.22	2.99	3.64	3.70	4.32	3.44
Trioxane	5.12	2.26	3.20	3.29	4.14	2.85
Urotropine	0.23	-0.34	-0.41	-0.42	-1.11	-0.49

Table S1: Relaxation energies, in kJ/mol, calculated for each method using the cc-pV5Z basis set, the crystal and the B3LYP optimized geometries. For CO_2 and Urotropine the relaxation energy is negative: this is somehow unusal, but it is related to the geometries optimized with a different method/basis-set as compared to the total energy calculation. As the relaxation error in those cases is much smaller than experimental error bar we can safely keep those negative relaxation energies.

S2 CBS extrapolation coefficients

System	SCF	E_x	MP2	W_∞	W'_∞	$W^{HF}_{1/2}$	E_{el}	$W^{HF}_{3/4}$
CO_2	3.58	2.95	1.59	3.15	3.91	0.97	2.49	0.38
NH_3	3.91	2.47*	1.79	4.06	5.20	3.08	3.71	0.87
Acetic acid	3.66	2.49	1.67	2.67	3.32	1.06	2.03	0.54
Cyanamide	3.65	2.45	1.65	3.52	4.59	1.77	2.14	0.47
Ethylcarb.	3.69	2.53	1.69	2.81	3.42	1.41	1.02	0.57
Ox. Acid β	3.61	2.36	1.64	2.12	2.81	1.06*	1.68	0.42
Succinic	3.60	2.61	1.66	2.61	3.19	0.59	1.86	0.49
Uracil	3.57	2.58	1.66	2.62	3.41	0.44	1.43	0.42
Cyclohex.	3.59	2.61	1.67	2.64	3.44	0.69	1.28	0.52
Formamide	3.64	2.57	1.68	3.07	3.95	1.60	2.32	0.55
Pyrazine	3.53	2.56	1.65	2.95	3.98	0.85	1.22	0.43
Triazine	3.50	2.40	1.64	2.71	3.76	0.79	1.11	0.41
Urea	3.66	1.69	1.69	2.66	3.76	1.15	1.75	0.54
Cytosine	3.59	2.36	1.67	2.64	3.65	0.62	1.19	0.43
Imidazole	3.56	2.42	1.67	2.76	3.68	0.87	1.22	0.46
Ox. acid α	3.61	2.32	1.64	2.11	2.83	1.06*	1.92	0.42
Pyrazole	3.55	2.27	1.66	2.59	3.51	0.49	0.84	0.46
Trioxane	3.75	2.89	1.68	2.34	2.13	0.63	1.95	0.54
Urotropine	3.56	1.98	1.69	2.03	2.57	0.57	0.46	0.54
Average	3.62	2.47	1.67	2.78	3.61	1.06	1.64	0.50

Table S2: Optimized CBS extrapolation coefficients for various systems and methods. (*) For E_x of NH_3 and $W^{HF}_{1/2}$ of both Ox. acids, the α could not be computed and thus it has been set to the averaged value.

S3 Benzene Crystal

We compute with CRYSTAL23 the HF energy and related ACM ingredients using the PBE-TS benzene crystal geometry as reported in Ref. 49 (of the main manuscript). Results are reported in Tab. S3 for the crystal and in Tab. S4 for the CPC system.

	cc-pVDZ	cc-pVTZ
HF	-922.86373214	-923.07718489
HF$^{(a)}$	-922.86201623	-923.07686768
E_x	-132.97652697	-133.02732815
W_∞	-215.11572508	-215.25171900
W'_∞	182.40317767	182.66838934
E_{el}	-287.77936811	-287.83633848
$W_{1/2}$	1027.67386340	1028.11287294
$W_{3/4}$	-630.09887014	-631.15911802
${E_{MP2}}^{(a)}$	-3.27710320	-3.95893940

$^{(a)}$ from Ref. 49

Table S3: ACM ingredients in the cc-pVDZ and cc-pVTZ for the benzene crystal (4 molecules in the unit cell).

	cc-pVDZ	cc-pVTZ
HF	-2.307247606297E+02	-2.307786683609E+02
HF$^{(a)}$	-230.7249620110	-230.7787316361
E_x	-3.320646950230E+01	-3.322026968545E+01
W_∞	-0.534894719341E+02	-0.535264817088E+02
W'_∞	0.452117248630E+02	0.452809186937E+02
E_{el}	-0.719539774799E+02	-0.719703904435E+02
$W_{1/2}$	0.256738658763E+03	0.256837483082E+03
$W_{3/4}$	-157.5258296142	-157.7922460278
${E_{MP2}}^{(a)}$	-0.7914804395	-0.954773982

$^{(a)}$ from Ref. 49

Table S4: ACM ingredients in the cc-pVDZ and cc-pVTZ for the benzene atom+ghosts CPC system (12 atoms + 74 ghosts).

Then we extrapolated that quantities using the procedure described in the manuscript. For MP2 we use $\alpha = 2.5$ which reproduces the CBS results in Ref. 49. For other ingredients we used the averaged values in Tab. S2.

The results are reported in Tab. S5. These results give an indication of the performance of the various approaches, however, they cannot be considered very accurate as the cc-pVDZ basis-set is very small (and smaller than the paug-cc-VTZ used in the main manuscript) so that the quality of the DZ-TZ extrapolation is limited.

HF	80.8	79.0[a]
MP2	-22.4	-21.8[a]
ISI	-5.8	
revISI	-5.1	
MPACF1	-1.5	
HFAC24	-16.6	

[a] from Ref. 49.

Table S5: Lattice errors in kJ/mol for the benzene crystal at CBS limit

S4 Additional data on lattice energies

System	MP2	ISI	revISI	MPACF1	HFAC24	Exp.
CO_2	-32.76	-29.18	-29.12	-29.70	-29.39	-29.4
1,4-Cyclohexanedione	-99.66	-84.74	-84.10	-85.34	-90.88	-90.0
Pyrazine	-83.04	-64.89	-63.89	-62.97	-73.15	-64.3
Triazine	-74.04	-60.01	-59.28	-59.43	-65.65	-62.6
Trioxane	-65.21	-53.16	-52.56	-52.86	-56.94	-64.6
Urotropine	-100.38	-79.64	-78.60	-78.41	-88.62	-84.1
Cytosine	-180.55	-158.17	-156.89	-157.91	-166.87	-163.5
Imidazole	-103.23	-88.23	-87.44	-87.25	-94.64	-90.4
Pyrazole	-93.74	-79.46	-78.69	-78.34	-85.28	-78.8
Uracil	-151.88	-132.99	-131.94	-133.28	-139.53	-136.2
Acetic acid	-73.69	-66.14	-65.89	-67.35	-68.11	-73.6
Cyanamide	-92.53	-83.29	-82.87	-83.65	-86.31	-81.5
Ethylcarbamate	-90.47	-79.36	-78.94	-79.76	-83.08	-88.2
Formamide	-83.13	-75.54	-75.25	-76.35	-77.51	-81.1
NH_3	-38.87	-34.32	-34.22	-34.42	-35.48	-38.7
Oxalic acid α	-104.29	-95.14	-94.80	-96.99	-96.34	-98.8
Oxalic acid β	-103.79	-93.40	-92.93	-94.82	-95.06	-96.8
Succinic acid	-131.56	-115.78	-115.07	-117.30	-120.24	-130.1
Urea	-108.72	-100.04	-99.73	-101.38	-102.15	-102.1

Table S6: Total Lattice energies (kJ/mol) for various computational methods. The experimental reference is reported as well.

system	HF	MP2	ISI	revISI	MPACF1	HFAC24
CO2	25.71	-2.99	0.48	0.53	-0.04	0.26
1,4-Cyclohexanedione	89.29	-9.62	5.28	5.91	4.68	-0.95
Pyrazine	87.65	-18.65	-0.56	0.43	1.39	-8.90
Triazine	74.89	-11.23	2.70	3.42	3.30	-3.06
Trioxane	77.37	-0.63	11.44	12.03	11.74	7.62
Urotropine	108.92	-16.55	4.32	5.38	5.51	-5.19
Cytosine	119.20	-17.11	5.29	6.57	5.55	-3.58
imidazole	77.26	-12.81	2.18	2.97	3.15	-4.27
Pyrazole	69.97	-14.88	-0.63	0.13	0.49	-6.65
Uracil	102.70	-15.57	3.28	4.32	3.00	-3.58
AceticAcid	55.76	-0.09	7.46	7.70	6.25	5.51
Cyanamide	50.28	-10.95	-1.73	-1.32	-2.08	-4.72
ethylcarbamate	74.21	-2.44	8.72	9.15	8.32	5.08
formamide	50.89	-2.09	5.51	5.81	4.71	3.62
NH3	32.46	-0.54	4.12	4.23	4.03	3.15
OxalicAcidAlpha	62.56	-5.22	3.86	4.20	2.00	2.63
OxalicAcidBeta	66.72	-6.72	3.60	4.06	2.18	1.90
SuccinicAcid	106.96	-1.40	14.36	15.07	12.85	9.89
Urea	55.91	-6.77	1.97	2.28	0.61	-0.12
ME	73.09	-8.22	4.30	4.89	4.09	-0.07
MAE	73.09	8.22	4.60	5.03	4.31	4.24

Table S7: Lattice energy errors in kJ/mol using the averaged exponents for CBS extrapolation

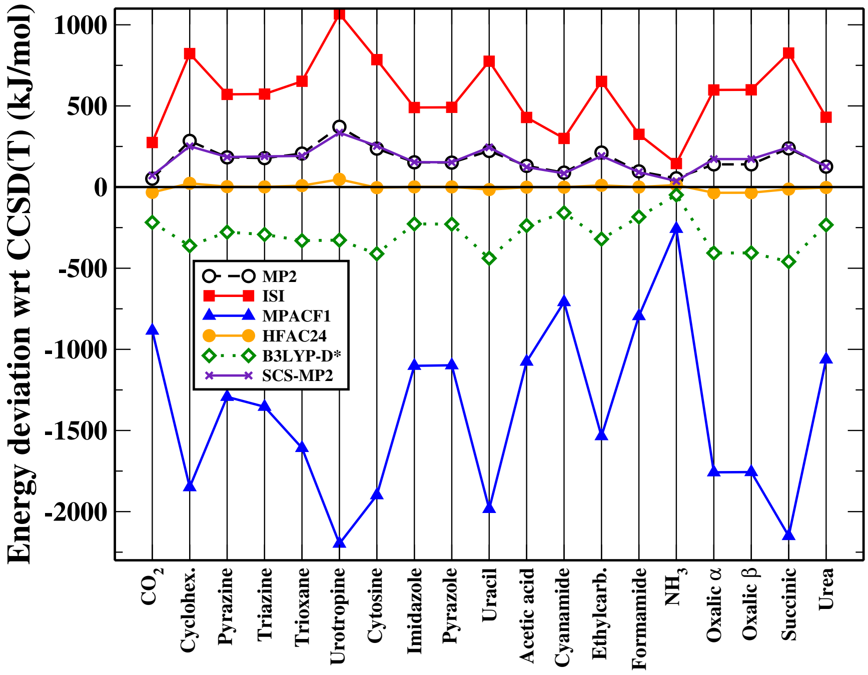

Figure S1: Total energy deviation in kJ/mol from the CCSD(T) one (in kJ/mol) for isolated molecules, as obtained with different methods. The revISI results are not reported because they overlap, on the scale of the plot, with the ISI ones.

S5 Non size-extensivity of the SPL2 model

The SPL2 correlation is defined by the formula

$$E_c = \int_0^1 d\lambda W_{c,\lambda}^{\mathrm{SPL2}} \tag{S1}$$

$$W_{c,\lambda}^{\mathrm{SPL2}} = C_1 - \frac{m_1}{\sqrt{1+b_1\lambda}} - \frac{m_2}{\sqrt{1+b_2\lambda}}\ , \tag{S2}$$

with

$$C_1 = W_{\alpha,\beta}^{\infty} \equiv \alpha W_{\infty}^{\mathrm{PC}} + \beta E_x \tag{S3}$$

$$b_1 = \frac{b_2 m_2 - 4E_c^{\mathrm{MP2}}}{m_2 - W_{\alpha,\beta}^{\infty}} \tag{S4}$$

$$m_1 = W_{\alpha,\beta}^{\infty} - m_2\ , \tag{S5}$$

and the parameters b_2, m_2, α, and β are empirically fixed.

For any integer N, we have that $W_{\alpha,\beta}^{\infty}(NW_{\infty}^{\mathrm{PC}}, NE_x) = NW_{\alpha,\beta}^{\infty}(W_{\infty}^{\mathrm{PC}}, E_x)$ and the same occurs for C_1. However, because b_2 and m_2 are fixed numbers, this same property in not valid for b_1 and m_1. Hence, the SPL2 formula is not size-extensive.

The size-extensivity is a fundamental property for periodic systems in which the following relation must hold

$$E_{cell}[M] = M * E_{cell} \tag{S6}$$

where M indicates the folding on the unit cell. Eq. (S6) must hold because the unit cell in a periodic solid can be defined in different ways. Eq. S6 is clearly violated by SPL2.

Then, if we consider the binding energy

$$E_b[M] = (E_{cell}[M]/M - E_{mol}) \tag{S7}$$

or with the SCC correction

$$E_b^{SCC}[M] = (E_{cell}[M] - E_{mol}^{SCC}[M])/M \tag{S8}$$

we have that both of them depend on M if the functional is not size-extensive.

In Fig. S2 we report $E_b[M]$ and $E_b^{SCC}[M]$ for one system (e.g. urotropine). Without SCC, the binding energy explodes already for M=2. With SCC the binding energy seems reliable but yet depends on M, i.e. it cannot be determined univocally.

In Tab. S8 we report the MAE and ME of the SPL2 total binding energy for all the systems investigated in the manuscript.

When $M \to \infty$ then the dependence on M will vanish because, in this case, the role of b_2 and m_2 parameters will vanish, and the SPL2 functional becomes size-extensive.

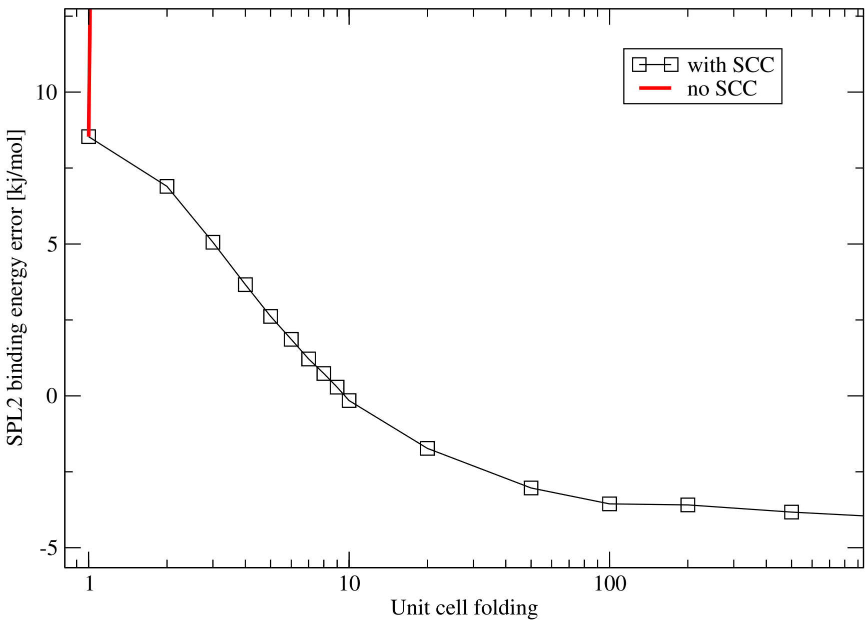

Figure S2: SPL2 binding energy error (in kJ/mol) for urotropine as a function of the unit-cell folding.

M	MAE	ME
1	4.15668	2.76226
2	3.37368	-0.13874
4	3.95884	-2.93884
8	5.35232	-5.00705
100	7.62047	-7.57511
1000	7.83147	-7.81074
10000	7.85379	-7.83526

Table S8: MAE and ME in kJ/mol for SPL2 for all the 19 systems investigated in the manuscript.

S6 Geometries

```
******
CO2
******

--- crystal

 LATTICE PARAMETERS (ANGSTROMS AND DEGREES) - BOHR = 0.5291772083 ANGSTROM
 PRIMITIVE CELL - CENTRING CODE 1/0 VOLUME=   172.589807 - DENSITY  1.693 g/cm^3
         A              B              C           ALPHA      BETA       GAMMA
     5.56764728     5.56764728     5.56764728    90.000000  90.000000  90.000000
 *******************************************************************************
 ATOMS IN THE ASYMMETRIC UNIT    2 - ATOMS IN THE UNIT CELL:   12
     ATOM                 X/A                 Y/B                 Z/C
 *******************************************************************************
      1 T   6 C     0.000000000000E+00  0.000000000000E+00  0.000000000000E+00
      2 F   6 C     5.000000000000E-01  0.000000000000E+00  5.000000000000E-01
      3 F   6 C     5.000000000000E-01  5.000000000000E-01  0.000000000000E+00
      4 F   6 C     0.000000000000E+00  5.000000000000E-01  5.000000000000E-01
      5 T   8 O     1.202096837265E-01  1.202096837265E-01  1.202096837265E-01
      6 F   8 O     3.797903162735E-01 -1.202096837265E-01 -3.797903162735E-01
      7 F   8 O    -3.797903162735E-01  3.797903162735E-01 -1.202096837265E-01
      8 F   8 O    -1.202096837265E-01 -3.797903162735E-01  3.797903162735E-01
      9 F   8 O    -1.202096837265E-01 -1.202096837265E-01 -1.202096837265E-01
     10 F   8 O    -3.797903162735E-01  1.202096837265E-01  3.797903162735E-01
     11 F   8 O     3.797903162735E-01 -3.797903162735E-01  1.202096837265E-01
     12 F   8 O     1.202096837265E-01  3.797903162735E-01 -3.797903162735E-01


--- molecule


      1 T   6 C     0.000000000000E+00  0.000000000000E+00  0.000000000000E+00
      2 T   8 O     6.701011830486E-01  6.701011830486E-01  6.701011830486E-01
      3 T   8 O    -6.701011830486E-01 -6.701011830486E-01 -6.701011830486E-01


******
NH3
******

--- crystal

*******************************************************************************
 LATTICE PARAMETERS (ANGSTROMS AND DEGREES) - BOHR = 0.5291772083 ANGSTROM
```

```
 PRIMITIVE CELL - CENTRING CODE 1/0 VOLUME=   124.223237 - DENSITY  0.910 g/cm^3
         A              B              C           ALPHA      BETA       GAMMA
    4.98962163     4.98962163     4.98962163    90.000000  90.000000  90.000000
 *******************************************************************************
 ATOMS IN THE ASYMMETRIC UNIT    2 - ATOMS IN THE UNIT CELL:   16
     ATOM                 X/A                 Y/B                 Z/C
 *******************************************************************************
      1 T   7 N     2.084099250349E-01  2.084099250349E-01  2.084099250349E-01
      2 F   7 N     2.915900749651E-01 -2.084099250349E-01 -2.915900749651E-01
      3 F   7 N    -2.915900749651E-01  2.915900749651E-01 -2.084099250349E-01
      4 F   7 N    -2.084099250349E-01 -2.915900749651E-01  2.915900749651E-01
      5 T   1 H     3.728071026608E-01  2.731138330800E-01  1.062181664284E-01
      6 F   1 H     1.271928973392E-01 -2.731138330800E-01 -3.937818335716E-01
      7 F   1 H    -1.271928973392E-01  2.268861669200E-01 -1.062181664284E-01
      8 F   1 H    -3.728071026608E-01 -2.268861669200E-01  3.937818335716E-01
      9 F   1 H     1.062181664284E-01  3.728071026608E-01  2.731138330800E-01
     10 F   1 H     2.731138330800E-01  1.062181664284E-01  3.728071026608E-01
     11 F   1 H     3.937818335716E-01 -3.728071026608E-01 -2.268861669200E-01
     12 F   1 H    -2.731138330800E-01 -3.937818335716E-01  1.271928973392E-01
     13 F   1 H    -3.937818335716E-01  1.271928973392E-01 -2.731138330800E-01
     14 F   1 H     2.268861669200E-01 -1.062181664284E-01 -1.271928973392E-01
     15 F   1 H    -1.062181664284E-01 -1.271928973392E-01  2.268861669200E-01
     16 F   1 H    -2.268861669200E-01  3.937818335716E-01 -3.728071026608E-01


--- molecule

      1 T   7 N     1.031472237839E+00  1.031472237839E+00  1.031472237839E+00
      2 T   1 H     1.855042302229E+00  1.364084772897E+00  5.421768898472E-01
      3 T   1 H     5.421768898472E-01  1.855042302229E+00  1.364084772897E+00
      4 T   1 H     1.364084772897E+00  5.421768898472E-01  1.855042302229E+00

******
Urea
******

--- crystal
*******************************************************************************
 LATTICE PARAMETERS (ANGSTROMS AND DEGREES) - BOHR = 0.5291772083 ANGSTROM
 PRIMITIVE CELL - CENTRING CODE 1/0 VOLUME=   143.538446 - DENSITY  1.389 g/cm^3
         A              B              C           ALPHA      BETA       GAMMA
    5.54245312     5.54245312     4.67266003    90.000000  90.000000  90.000000
 *******************************************************************************
 ATOMS IN THE ASYMMETRIC UNIT    5 - ATOMS IN THE UNIT CELL:   16
```

```
    ATOM                 X/A                 Y/B                 Z/C
 *******************************************************************************
      1 T   6 C    -7.724318686835E-18 -5.000000000000E-01  3.194785654647E-01
      2 F   6 C    -5.000000000000E-01 -1.110223024625E-16 -3.194785654647E-01
      3 T   8 O    -7.724318686835E-18 -5.000000000000E-01 -4.098919778825E-01
      4 F   8 O    -5.000000000000E-01 -1.110223024625E-16  4.098919778825E-01
      5 T   7 N     1.468121950316E-01 -3.531878049684E-01  1.708031608317E-01
      6 F   7 N    -1.468121950316E-01  3.531878049684E-01  1.708031608317E-01
      7 F   7 N    -3.531878049684E-01 -1.468121950316E-01 -1.708031608317E-01
      8 F   7 N     3.531878049684E-01  1.468121950316E-01 -1.708031608317E-01
      9 T   1 H     2.594816054246E-01 -2.405183945754E-01  2.768858341994E-01
     10 F   1 H    -2.594816054246E-01  2.405183945754E-01  2.768858341994E-01
     11 F   1 H    -2.405183945754E-01 -2.594816054246E-01 -2.768858341994E-01
     12 F   1 H     2.405183945754E-01  2.594816054246E-01 -2.768858341994E-01
     13 T   1 H     1.464728420823E-01 -3.535271579177E-01 -4.572292800875E-02
     14 F   1 H    -1.464728420823E-01  3.535271579177E-01 -4.572292800875E-02
     15 F   1 H    -3.535271579177E-01 -1.464728420823E-01  4.572292800875E-02
     16 F   1 H     3.535271579177E-01  1.464728420823E-01  4.572292800875E-02


--- molecule

      1 T   6 C    -3.298534756769E-05 -2.771189676137E+00  1.524261070575E+00
      2 T   8 O     1.275175258424E-04 -2.771352228876E+00  2.742250920442E+00
      3 T   7 N     8.195237394258E-01 -1.951659193678E+00  7.873281189569E-01
      4 T   7 N    -8.195653701865E-01 -3.590757796295E+00  7.873239182573E-01
      5 T   1 H     8.389204639778E-01 -1.932389512628E+00 -2.145802361302E-01
      6 T   1 H     1.429696521529E+00 -1.341375769844E+00  1.297343042307E+00
      7 T   1 H    -8.387892393935E-01 -3.610194547863E+00 -2.145792410300E-01
      8 T   1 H    -1.429880647531E+00 -4.200893754679E+00  1.297342847824E+00

******
Trioxane
******

--- crystal


*******************************************************************************
 LATTICE PARAMETERS (ANGSTROMS AND DEGREES) - BOHR = 0.5291772083 ANGSTROM
 PRIMITIVE CELL - CENTRING CODE 7/0 VOLUME=   193.594371 - DENSITY  1.544 g/cm^3
         A              B              C           ALPHA      BETA       GAMMA
     5.94362911     5.94362911     5.94362911   102.086460 102.086460 102.086460
 *******************************************************************************
```

```
 ATOMS IN THE ASYMMETRIC UNIT    4 - ATOMS IN THE UNIT CELL:   24
     ATOM                 X/A                 Y/B                 Z/C
 *******************************************************************************
      1 T   6 C     4.935127767024E-01 -3.902356770582E-01  3.207429343399E-01
      2 F   6 C     3.207429343399E-01  4.935127767024E-01 -3.902356770582E-01
      3 F   6 C    -3.902356770582E-01  3.207429343399E-01  4.935127767024E-01
      4 F   6 C     1.097643229418E-01 -6.487223297550E-03 -1.792570656601E-01
      5 F   6 C    -6.487223297550E-03 -1.792570656601E-01  1.097643229418E-01
      6 F   6 C    -1.792570656601E-01  1.097643229418E-01 -6.487223297550E-03
      7 T   1 H    -4.372570491436E-01 -2.349759356706E-01  2.628381748264E-01
      8 F   1 H     2.628381748264E-01 -4.372570491436E-01 -2.349759356706E-01
      9 F   1 H    -2.349759356706E-01  2.628381748264E-01 -4.372570491436E-01
     10 F   1 H     2.650240643294E-01  6.274295085640E-02 -2.371618251736E-01
     11 F   1 H     6.274295085640E-02 -2.371618251736E-01  2.650240643294E-01
     12 F   1 H    -2.371618251736E-01  2.650240643294E-01  6.274295085640E-02
     13 T   1 H     3.542525688938E-01  4.744044709249E-01  1.769577870977E-01
     14 F   1 H     1.769577870977E-01  3.542525688938E-01  4.744044709249E-01
     15 F   1 H     4.744044709249E-01  1.769577870977E-01  3.542525688938E-01
     16 F   1 H    -2.559552907505E-02 -1.457474311062E-01 -3.230422129023E-01
     17 F   1 H    -1.457474311062E-01 -3.230422129023E-01 -2.559552907505E-02
     18 F   1 H    -3.230422129023E-01 -2.559552907505E-02 -1.457474311061E-01
     19 T   8 O     1.870724865669E-01 -1.024649404600E-01  1.415240298017E-02
     20 F   8 O     1.415240298017E-02  1.870724865669E-01 -1.024649404600E-01
     21 F   8 O    -1.024649404600E-01  1.415240298017E-02  1.870724865669E-01
     22 F   8 O     3.975350595400E-01 -3.129275134331E-01 -4.858475970198E-01
     23 F   8 O    -3.129275134331E-01 -4.858475970198E-01  3.975350595400E-01
     24 F   8 O    -4.858475970198E-01  3.975350595400E-01 -3.129275134331E-01

--- molecule

      1 T   6 C     1.492111045898E-01  1.329800490018E+00  3.729648713235E+00
      2 T   1 H     2.594285783860E-01  2.313957032128E+00  4.174936195602E+00
      3 T   1 H     1.564335019654E-01  1.395905605701E+00  2.628351984052E+00
      4 T   8 O     1.235759005428E+00  5.400037163344E-01  4.167229735991E+00
      5 T   8 O    -1.085536439197E+00  8.001968334885E-01  4.167229735991E+00
      6 T   6 C     1.077035454025E+00 -7.941208521104E-01  3.729648713235E+00
      7 T   6 C    -1.226246558615E+00 -5.356796379074E-01  3.729648713235E+00
      8 T   1 H     1.874231283896E+00 -1.381650255414E+00  4.174936195602E+00
      9 T   1 H     1.130672964840E+00 -8.334281895556E-01  2.628351984052E+00
     10 T   8 O    -1.502225662302E-01 -1.340200549823E+00  4.167229735991E+00
     11 T   1 H    -2.133659862282E+00 -9.323067767142E-01  4.174936195602E+00
     12 T   1 H    -1.287106466805E+00 -5.624774161456E-01  2.628351984052E+00
```

```
******
Urotropine
******

--- crystal

*******************************************************************************
 LATTICE PARAMETERS (ANGSTROMS AND DEGREES) - BOHR = 0.5291772083 ANGSTROM
 PRIMITIVE CELL - CENTRING CODE 6/0 VOLUME=   162.732994 - DENSITY  1.430 g/cm^3
         A              B              C           ALPHA      BETA       GAMMA
     5.95706724     5.95706724     5.95706724   109.471221 109.471221 109.471221
 *******************************************************************************
 ATOMS IN THE ASYMMETRIC UNIT    3 - ATOMS IN THE UNIT CELL:   22
     ATOM                 X/A                 Y/B                 Z/C
 *******************************************************************************
      1 T   6 C     0.000000000000E+00  2.458162872307E-01  2.458162872307E-01
      2 F   6 C     0.000000000000E+00 -2.458162872307E-01 -2.458162872307E-01
      3 F   6 C     2.458162872307E-01  0.000000000000E+00  2.458162872307E-01
      4 F   6 C     2.458162872307E-01  2.458162872307E-01  0.000000000000E+00
      5 F   6 C    -2.458162872307E-01  0.000000000000E+00 -2.458162872307E-01
      6 F   6 C    -2.458162872307E-01 -2.458162872307E-01  0.000000000000E+00
      7 T   7 N     2.520329383814E-01  2.520329383814E-01  2.520329383814E-01
      8 F   7 N     0.000000000000E+00  2.775557561563E-17 -2.520329383814E-01
      9 F   7 N    -2.520329383814E-01 -2.775557561563E-17 -2.775557561563E-17
     10 F   7 N     0.000000000000E+00 -2.520329383814E-01  2.775557561563E-17
     11 T   1 H    -2.449372432127E-01 -2.449372432127E-01  1.834800676911E-01
     12 F   1 H    -4.284173109039E-01 -4.284173109039E-01 -1.834800676911E-01
     13 F   1 H     2.449372432127E-01  4.284173109039E-01 -2.775557561563E-17
     14 F   1 H     4.284173109039E-01  2.449372432127E-01  4.163336342344E-17
     15 F   1 H     1.834800676911E-01 -2.449372432127E-01 -2.449372432127E-01
     16 F   1 H    -2.449372432127E-01  1.834800676911E-01 -2.449372432127E-01
     17 F   1 H     6.938893903907E-17  4.284173109039E-01  2.449372432127E-01
     18 F   1 H    -4.284173109039E-01 -1.834800676911E-01 -4.284173109039E-01
     19 F   1 H    -1.834800676911E-01 -4.284173109039E-01 -4.284173109039E-01
     20 F   1 H     4.284173109039E-01 -6.938893903907E-17  2.449372432127E-01
     21 F   1 H    -6.938893903907E-17  2.449372432127E-01  4.284173109039E-01
     22 F   1 H     2.449372432127E-01  4.163336342344E-17  4.284173109039E-01

--- molecule

      1 T   1 H    -1.986567862534E+00  2.170250139238E+00  3.759051302762E+00
      2 T   1 H    -3.430129963193E+00  1.659857582256E+00  2.875053133627E+00
      3 T   1 H    -5.398826113067E+00  9.638665427825E-01  4.080613139267E+00
      4 T   1 H    -5.398832220242E+00  9.638688165252E-01  5.848595834241E+00
```

```
     5 T   1 H    -1.986557426238E+00  2.170260523396E+00  6.170140779850E+00
     6 T   1 H    -3.430105464047E+00  1.659865873304E+00  7.054166109487E+00
     7 T   1 H    -5.398848619173E+00  3.052012488726E+00  2.874985264488E+00
     8 T   1 H    -5.398775842742E+00  4.583101311261E+00  3.759012331002E+00
     9 T   1 H    -1.986593370373E+00  4.258478680829E+00  4.964581003045E+00
    10 T   1 H    -3.430218521790E+00  5.279142200353E+00  4.964590484134E+00
    11 T   1 H    -5.398837136826E+00  3.051984294258E+00  7.054235330328E+00
    12 T   1 H    -5.398770588883E+00  4.583093862566E+00  6.170222089369E+00
    13 T   6 C    -3.078499061268E+00  2.176813159710E+00  3.770380240876E+00
    14 T   6 C    -5.028655579969E+00  1.487351265721E+00  4.964604687673E+00
    15 T   6 C    -3.078490479548E+00  2.176816703511E+00  6.158829966901E+00
    16 T   6 C    -5.028646859128E+00  3.555787262349E+00  3.770344093603E+00
    17 T   6 C    -3.078530510557E+00  4.245305314404E+00  4.964601734926E+00
    18 T   6 C    -5.028635771000E+00  3.555780733088E+00  6.158884803796E+00
    19 T   7 N    -3.552487251030E+00  1.448967836226E+00  4.964607667264E+00
    20 T   7 N    -3.552470107627E+00  3.574991290328E+00  3.737099457315E+00
    21 T   7 N    -5.556956593922E+00  2.866315837840E+00  4.964604953857E+00
    22 T   7 N    -3.552464656844E+00  3.574988281326E+00  6.192095592187E+00

******
Pyrazine
******

--- crystal

*******************************************************************************
 LATTICE PARAMETERS (ANGSTROMS AND DEGREES) - BOHR = 0.5291772083 ANGSTROM
 PRIMITIVE CELL - CENTRING CODE 1/0 VOLUME=   191.482852 - DENSITY  1.388 g/cm^3
         A              B              C           ALPHA      BETA       GAMMA
     9.25053832     5.64151569     3.66916364    90.000000  90.000000  90.000000
 *******************************************************************************
 ATOMS IN THE ASYMMETRIC UNIT    3 - ATOMS IN THE UNIT CELL:   20
     ATOM                 X/A                 Y/B                 Z/C
 *******************************************************************************
     1 T   7 N     1.508653680197E-01 -3.413851968543E-35  0.000000000000E+00
     2 F   7 N     3.491346319803E-01 -5.000000000000E-01  5.000000000000E-01
     3 F   7 N    -1.508653680197E-01  3.413851968543E-35  0.000000000000E+00
     4 F   7 N    -3.491346319803E-01 -5.000000000000E-01  5.000000000000E-01
     5 T   6 C     7.522973767188E-02  1.817603207902E-01  1.338790234924E-01
     6 F   6 C     4.247702623281E-01  3.182396792098E-01 -3.661209765076E-01
     7 F   6 C     7.522973767188E-02 -1.817603207902E-01 -1.338790234924E-01
     8 F   6 C     4.247702623281E-01 -3.182396792098E-01  3.661209765076E-01
     9 F   6 C    -7.522973767188E-02 -1.817603207902E-01 -1.338790234924E-01
    10 F   6 C    -4.247702623281E-01 -3.182396792098E-01  3.661209765076E-01
```

```
    11 F   6 C    -7.522973767188E-02  1.817603207902E-01  1.338790234924E-01
    12 F   6 C    -4.247702623281E-01  3.182396792098E-01 -3.661209765076E-01
    13 T   1 H     1.366698205476E-01  3.282314260154E-01  2.458436822577E-01
    14 F   1 H     3.633301794524E-01  1.717685739846E-01 -2.541563177423E-01
    15 F   1 H     1.366698205476E-01 -3.282314260154E-01 -2.458436822577E-01
    16 F   1 H     3.633301794524E-01 -1.717685739846E-01  2.541563177423E-01
    17 F   1 H    -1.366698205476E-01 -3.282314260154E-01 -2.458436822577E-01
    18 F   1 H    -3.633301794524E-01 -1.717685739846E-01  2.541563177423E-01
    19 F   1 H    -1.366698205476E-01  3.282314260154E-01  2.458436822577E-01
    20 F   1 H    -3.633301794524E-01  1.717685739846E-01 -2.541563177423E-01
```

--- molecule

```
     1 T   7 N     1.403536952019E+00  2.781040958785E-19  1.272038235034E-19
     2 T   6 C     6.967789267561E-01  1.017720531743E+00  4.938630845288E-01
     3 T   6 C     6.967789267561E-01 -1.017720531743E+00 -4.938630845288E-01
     4 T   1 H     1.254693676607E+00  1.854600320424E+00  8.999429002214E-01
     5 T   6 C    -6.967789267561E-01  1.017720531743E+00  4.938630845288E-01
     6 T   1 H     1.254693676607E+00 -1.854600320424E+00 -8.999429002214E-01
     7 T   6 C    -6.967789267561E-01 -1.017720531743E+00 -4.938630845288E-01
     8 T   1 H    -1.254693676607E+00  1.854600320424E+00  8.999429002214E-01
     9 T   7 N    -1.403536952019E+00 -3.771508896920E-19 -2.743859694452E-19
    10 T   1 H    -1.254693676607E+00 -1.854600320424E+00 -8.999429002214E-01
```

******
Formamide
******

--- crystal

```
******************************************************************************
 LATTICE PARAMETERS (ANGSTROMS AND DEGREES) - BOHR = 0.5291772083 ANGSTROM
 PRIMITIVE CELL - CENTRING CODE 1/0 VOLUME=   216.468210 - DENSITY  1.381 g/cm^3
         A              B              C           ALPHA      BETA       GAMMA
    3.50866157     9.03149800     6.96910466    90.000000 101.419848  90.000000
 *******************************************************************************
 ATOMS IN THE ASYMMETRIC UNIT    6 - ATOMS IN THE UNIT CELL:   24
    ATOM                 X/A                 Y/B                 Z/C
 *******************************************************************************
     1 T   8 O     4.799874643208E-01 -6.931027203040E-02  2.493406972572E-01
     2 F   8 O     2.001253567920E-02  4.306897279696E-01  2.506593027428E-01
     3 F   8 O    -4.799874643208E-01  6.931027203040E-02 -2.493406972572E-01
     4 F   8 O    -2.001253567920E-02 -4.306897279696E-01 -2.506593027428E-01
```

```
     5 T   7 N     2.828678156071E-01  1.553216329818E-01  1.162139285564E-01
     6 F   7 N     2.171321843929E-01 -3.446783670182E-01  3.837860714436E-01
     7 F   7 N    -2.828678156071E-01 -1.553216329818E-01 -1.162139285564E-01
     8 F   7 N    -2.171321843929E-01  3.446783670182E-01 -3.837860714436E-01
     9 T   6 C     3.300433598305E-01  5.493155347728E-02  2.569945364521E-01
    10 F   6 C     1.699566401695E-01 -4.450684465227E-01  2.430054635479E-01
    11 F   6 C    -3.300433598305E-01 -5.493155347728E-02 -2.569945364521E-01
    12 F   6 C    -1.699566401695E-01  4.450684465227E-01 -2.430054635479E-01
    13 T   1 H     1.737175735825E-01  2.564985814244E-01  1.418113460816E-01
    14 F   1 H     3.262824264175E-01 -2.435014185756E-01  3.581886539184E-01
    15 F   1 H    -1.737175735825E-01 -2.564985814244E-01 -1.418113460816E-01
    16 F   1 H    -3.262824264175E-01  2.435014185756E-01 -3.581886539184E-01
    17 T   1 H     3.632357900348E-01  1.325570319678E-01 -1.398771413720E-02
    18 F   1 H     1.367642099652E-01 -3.674429680322E-01 -4.860122858628E-01
    19 F   1 H    -3.632357900348E-01 -1.325570319678E-01  1.398771413720E-02
    20 F   1 H    -1.367642099652E-01  3.674429680322E-01  4.860122858628E-01
    21 T   1 H     2.289489722059E-01  8.931062552042E-02  3.891717120018E-01
    22 F   1 H     2.710510277941E-01 -4.106893744796E-01  1.108282879982E-01
    23 F   1 H    -2.289489722059E-01 -8.931062552042E-02 -3.891717120018E-01
    24 F   1 H    -2.710510277941E-01  4.106893744796E-01 -1.108282879982E-01

--- molecule

     1 T   8 O     1.319931702741E+00 -6.295666723393E-01  1.689339805466E+00
     2 T   6 C     8.160152120725E-01  4.683576017455E-01  1.772981482763E+00
     3 T   1 H     2.782232899052E-01  8.116220221937E-01  2.676064301465E+00
     4 T   7 N     8.361384243288E-01  1.414306879978E+00  7.963244920821E-01
     5 T   1 H     3.994415347874E-01  2.310970585371E+00  9.271152931564E-01
     6 T   1 H     1.299740171876E+00  1.217598962834E+00 -7.744322049038E-02


******
Triazine
******

--- crystal

*******************************************************************************
 LATTICE PARAMETERS (ANGSTROMS AND DEGREES) - BOHR = 0.5291772083 ANGSTROM
 PRIMITIVE CELL - CENTRING CODE 7/0 VOLUME=   177.704054 - DENSITY  1.514 g/cm^3
        A              B              C           ALPHA      BETA       GAMMA
    5.93402615     5.93402615     5.93402615   106.194285 106.194285 106.194285
 *******************************************************************************
 ATOMS IN THE ASYMMETRIC UNIT    3 - ATOMS IN THE UNIT CELL:   18
```

```
     ATOM                 X/A                 Y/B                 Z/C
 *******************************************************************************
      1 T   6 C     2.500000000000E-01  3.868809659664E-01  1.131190340336E-01
      2 F   6 C     1.131190340336E-01  2.500000000000E-01  3.868809659664E-01
      3 F   6 C     3.868809659664E-01  1.131190340336E-01  2.500000000000E-01
      4 F   6 C    -2.500000000000E-01 -3.868809659664E-01 -1.131190340336E-01
      5 F   6 C    -1.131190340336E-01 -2.500000000000E-01 -3.868809659664E-01
      6 F   6 C    -3.868809659664E-01 -1.131190340336E-01 -2.500000000000E-01
      7 T   1 H     2.500000000000E-01 -4.990927983604E-01 -9.072016396000E-04
      8 F   1 H    -9.072016396000E-04  2.500000000000E-01 -4.990927983604E-01
      9 F   1 H    -4.990927983604E-01 -9.072016396000E-04  2.500000000000E-01
     10 F   1 H    -2.500000000000E-01  4.990927983604E-01  9.072016396000E-04
     11 F   1 H     9.072016396000E-04 -2.500000000000E-01  4.990927983604E-01
     12 F   1 H     4.990927983604E-01  9.072016396000E-04 -2.500000000000E-01
     13 T   7 N    -2.500000000000E-01 -1.060097605702E-01 -3.939902394298E-01
     14 F   7 N    -3.939902394298E-01 -2.500000000000E-01 -1.060097605702E-01
     15 F   7 N    -1.060097605702E-01 -3.939902394298E-01 -2.500000000000E-01
     16 F   7 N     2.500000000000E-01  1.060097605702E-01  3.939902394298E-01
     17 F   7 N     3.939902394298E-01  2.500000000000E-01  1.060097605702E-01
     18 F   7 N     1.060097605702E-01  3.939902394298E-01  2.500000000000E-01


--- molecule

      1 T   6 C    -3.561359866374E-07  1.293560868077E+00  1.708693270743E+00
      2 T   1 H     4.240944043234E-07  2.378235338411E+00  1.708691483616E+00
      3 T   7 N     1.186247796091E+00  6.848809467922E-01  1.708694508141E+00
      4 T   7 N    -1.186248196535E+00  6.848802532019E-01  1.708694508141E+00
      5 T   6 C     1.120256751164E+00 -6.467801256157E-01  1.708693270743E+00
      6 T   6 C    -1.120256395028E+00 -6.467807424613E-01  1.708693270743E+00
      7 T   1 H     2.059612007195E+00 -1.189118036482E+00  1.708691483616E+00
      8 T   7 N     4.004445204494E-07 -1.369761199994E+00  1.708694508141E+00
      9 T   1 H    -2.059612431289E+00 -1.189117301929E+00  1.708691483616E+00

******
Ethylcarbamate
******

--- crystal

 *******************************************************************************
 LATTICE PARAMETERS (ANGSTROMS AND DEGREES) - BOHR = 0.5291772083 ANGSTROM
 PRIMITIVE CELL - CENTRING CODE 1/0 VOLUME=   234.251472 - DENSITY  1.262 g/cm^3
         A              B              C           ALPHA      BETA       GAMMA
```

```
    5.01851714     6.96618932     7.30358799   100.354170 107.578131  75.607212
*******************************************************************************
 ATOMS IN THE ASYMMETRIC UNIT   13 - ATOMS IN THE UNIT CELL:   26
     ATOM                 X/A                 Y/B                 Z/C
*******************************************************************************
      1 T   6 C    -1.206393932705E-01 -2.032420992484E-01  3.662174466170E-01
      2 F   6 C     1.206393932705E-01  2.032420992484E-01 -3.662174466170E-01
      3 T   6 C    -2.074853051270E-01 -3.984703044276E-01  2.744489427887E-01
      4 F   6 C     2.074853051270E-01  3.984703044276E-01 -2.744489427887E-01
      5 T   6 C     1.905749190503E-02  2.789931581380E-01  1.553522686395E-01
      6 F   6 C    -1.905749190503E-02 -2.789931581380E-01 -1.553522686395E-01
      7 T   1 H    -3.029364398850E-01 -9.739883381120E-02  4.019881654792E-01
      8 F   1 H     3.029364398850E-01  9.739883381120E-02 -4.019881654792E-01
      9 T   1 H     5.143244786372E-02 -2.222825215230E-01  4.988754669804E-01
     10 F   1 H    -5.143244786372E-02  2.222825215230E-01 -4.988754669804E-01
     11 T   1 H    -4.989869519503E-02 -1.384743810724E-01  2.686355129873E-01
     12 F   1 H     4.989869519503E-02  1.384743810724E-01 -2.686355129873E-01
     13 T   1 H    -2.855956430573E-01 -4.603905594721E-01  3.703654646051E-01
     14 F   1 H     2.855956430573E-01  4.603905594721E-01 -3.703654646051E-01
     15 T   1 H    -3.726716645068E-01 -3.820855814113E-01  1.383533549550E-01
     16 F   1 H     3.726716645068E-01  3.820855814113E-01 -1.383533549550E-01
     17 T   1 H     4.441204756974E-01  2.166039637129E-01  1.631506542578E-01
     18 F   1 H    -4.441204756974E-01 -2.166039637129E-01 -1.631506542578E-01
     19 T   1 H     2.608139045998E-01  2.305671324477E-02  6.499975951723E-02
     20 F   1 H    -2.608139045998E-01 -2.305671324477E-02 -6.499975951723E-02
     21 T   7 N     2.675630097734E-01  1.602134158228E-01  1.405468724193E-01
     22 F   7 N    -2.675630097734E-01 -1.602134158228E-01 -1.405468724193E-01
     23 T   8 O     4.673341043372E-02  4.634425155201E-01  2.388760160519E-01
     24 F   8 O    -4.673341043372E-02 -4.634425155201E-01 -2.388760160519E-01
     25 T   8 O    -2.115790099203E-01  2.245398223155E-01  1.017888254461E-01
     26 F   8 O     2.115790099203E-01 -2.245398223155E-01 -1.017888254461E-01
```

```
--- molecule

      1 T   6 C    -1.289728808852E+00 -2.045426924833E+00  2.535288479643E+00
      2 T   1 H    -7.527971524650E-01 -2.145096496263E+00  3.480370831221E+00
      3 T   1 H    -7.089862532884E-01 -1.398745440442E+00  1.874906167234E+00
      4 T   1 H    -2.246674951219E+00 -1.558034166345E+00  2.735549198589E+00
      5 T   6 C    -1.530275985552E+00 -3.398532923704E+00  1.899829534349E+00
      6 T   1 H    -2.066000821793E+00 -3.311688148473E+00  9.522047025506E-01
      7 T   1 H    -2.110085348207E+00 -4.055012317552E+00  2.552104926496E+00
      8 T   8 O    -2.379110921622E-01 -3.999833092210E+00  1.656842771332E+00
      9 T   6 C    -2.460384500925E-01 -5.230532784967E+00  1.082295174239E+00
```

```
    10 T   8 O    -1.242289836674E+00 -5.836052787591E+00  7.565077283351E-01
    11 T   7 N     1.030665251375E+00 -5.689139749463E+00  9.481224541324E-01
    12 T   1 H     1.809774557968E+00 -5.079455568301E+00  1.125302366356E+00
    13 T   1 H     1.171766438130E+00 -6.534201905034E+00  4.238046330232E-01
```

```
******
Uracil
******

--- crystal

 *******************************************************************************
 LATTICE PARAMETERS (ANGSTROMS AND DEGREES) - BOHR = 0.5291772083 ANGSTROM
 PRIMITIVE CELL - CENTRING CODE 1/0 VOLUME=   441.083006 - DENSITY  1.687 g/cm^3
         A              B              C           ALPHA      BETA       GAMMA
    11.90096803    12.30848736     3.55213484    90.000000 122.037253  90.000000
 *******************************************************************************
 ATOMS IN THE ASYMMETRIC UNIT   12 - ATOMS IN THE UNIT CELL:   48
     ATOM                 X/A                 Y/B                 Z/C
 *******************************************************************************
      1 T   6 C     1.532446862733E-01  2.123776980744E-01  1.877423837152E-02
      2 F   6 C     3.467553137267E-01 -2.876223019256E-01 -1.877423837152E-02
      3 F   6 C    -1.532446862733E-01 -2.123776980744E-01 -1.877423837152E-02
      4 F   6 C    -3.467553137267E-01  2.876223019256E-01  1.877423837152E-02
      5 T   6 C     1.799860842874E-01  1.373830128686E-02  3.418515617274E-02
      6 F   6 C     3.200139157126E-01 -4.862616987131E-01 -3.418515617274E-02
      7 F   6 C    -1.799860842874E-01 -1.373830128686E-02 -3.418515617274E-02
      8 F   6 C    -3.200139157126E-01  4.862616987131E-01  3.418515617274E-02
      9 T   6 C     3.065635378869E-01  2.970459222972E-02  8.592643613033E-02
     10 F   6 C     1.934364621131E-01 -4.702954077703E-01 -8.592643613033E-02
     11 F   6 C    -3.065635378869E-01 -2.970459222972E-02 -8.592643613033E-02
     12 F   6 C    -1.934364621131E-01  4.702954077703E-01  8.592643613033E-02
     13 T   6 C     3.486708263596E-01  1.323426392603E-01  9.528043517165E-02
     14 F   6 C     1.513291736404E-01 -3.676573607397E-01 -9.528043517165E-02
     15 F   6 C    -3.486708263596E-01 -1.323426392603E-01 -9.528043517165E-02
     16 F   6 C    -1.513291736404E-01  3.676573607397E-01  9.528043517165E-02
     17 T   1 H     4.414161496207E-01  1.510631895179E-01  1.223409193899E-01
     18 F   1 H     5.858385037930E-02 -3.489368104821E-01 -1.223409193899E-01
     19 F   1 H    -4.414161496207E-01 -1.510631895179E-01 -1.223409193899E-01
     20 F   1 H    -5.858385037930E-02  3.489368104821E-01  1.223409193899E-01
     21 T   1 H     3.072293150906E-01  2.974858370430E-01  5.237122232548E-02
     22 F   1 H     1.927706849094E-01 -2.025141629570E-01 -5.237122232548E-02
     23 F   1 H    -3.072293150906E-01 -2.974858370430E-01 -5.237122232548E-02
```

```
  24 F   1 H    -1.927706849094E-01  2.025141629570E-01  5.237122232548E-02
  25 T   1 H     2.132639922027E-02  9.842728602065E-02 -2.188150995603E-02
  26 F   1 H     4.786736007797E-01 -4.015727139793E-01  2.188150995603E-02
  27 F   1 H    -2.132639922027E-02 -9.842728602065E-02  2.188150995603E-02
  28 F   1 H    -4.786736007797E-01  4.015727139794E-01 -2.188150995603E-02
  29 T   1 H     3.633226546114E-01 -4.043311574745E-02  1.020206470478E-01
  30 F   1 H     1.366773453886E-01  4.595668842526E-01 -1.020206470478E-01
  31 F   1 H    -3.633226546114E-01  4.043311574745E-02 -1.020206470478E-01
  32 F   1 H    -1.366773453886E-01 -4.595668842525E-01  1.020206470478E-01
  33 T   7 N     2.748372679098E-01  2.206215460351E-01  6.311905299333E-02
  34 F   7 N     2.251627320902E-01 -2.793784539649E-01 -6.311905299333E-02
  35 F   7 N    -2.748372679098E-01 -2.206215460351E-01 -6.311905299333E-02
  36 F   7 N    -2.251627320902E-01  2.793784539649E-01  6.311905299333E-02
  37 T   7 N     1.118691125750E-01  1.072035761940E-01  8.681188871695E-03
  38 F   7 N     3.881308874250E-01 -3.927964238060E-01 -8.681188871695E-03
  39 F   7 N    -1.118691125750E-01 -1.072035761940E-01 -8.681188871695E-03
  40 F   7 N    -3.881308874250E-01  3.927964238060E-01  8.681188871695E-03
  41 T   8 O     8.663211604596E-02  2.911174268735E-01 -7.470923035646E-03
  42 F   8 O     4.133678839540E-01 -2.088825731265E-01  7.470923035646E-03
  43 F   8 O    -8.663211604596E-02 -2.911174268735E-01  7.470923035646E-03
  44 F   8 O    -4.133678839540E-01  2.088825731265E-01 -7.470923035646E-03
  45 T   8 O     1.282976963784E-01 -7.620397978816E-02  1.062516168649E-02
  46 F   8 O     3.717023036216E-01  4.237960202118E-01 -1.062516168649E-02
  47 F   8 O    -1.282976963784E-01  7.620397978816E-02 -1.062516168649E-02
  48 F   8 O    -3.717023036216E-01 -4.237960202118E-01  1.062516168649E-02

--- molecule

   1 T   6 C     1.768279558107E+00  2.633291666358E+00  4.809193912997E-02
   2 T   8 O     1.055383183745E+00  3.609337530039E+00 -3.073787296109E-02
   3 T   7 N     3.153024382912E+00  2.718196331815E+00  1.781323006439E-01
   4 T   7 N     1.309774431480E+00  1.328013778569E+00  1.979254843830E-02
   5 T   1 H     3.525894614891E+00  3.654632708511E+00  2.023682934924E-01
   6 T   6 C     3.976695448112E+00  1.622581617675E+00  2.691052512222E-01
   7 T   1 H     3.080403787290E-01  1.217755071994E+00 -7.390343470585E-02
   8 T   6 C     2.063954891538E+00  1.362781004690E-01  1.049236537167E-01
   9 T   1 H     5.031289827909E+00  1.842975733546E+00  3.666676392703E-01
  10 T   6 C     3.497925285615E+00  3.661340003466E-01  2.386695810982E-01
  11 T   8 O     1.511649070492E+00 -9.439255152422E-01  6.473348841414E-02
  12 T   1 H     4.146441705847E+00 -4.924974478145E-01  3.103647401804E-01

******
Oxalix acid
******
```

--- crystal - alpha

```
*******************************************************************************
 LATTICE PARAMETERS (ANGSTROMS AND DEGREES) - BOHR = 0.5291772083 ANGSTROM
 PRIMITIVE CELL - CENTRING CODE 1/0 VOLUME=   306.326763 - DENSITY  1.951 g/cm^3
         A              B              C           ALPHA      BETA       GAMMA
     6.50760188     7.67236487     6.13528466    90.000000  90.000000  90.000000
 *******************************************************************************
 ATOMS IN THE ASYMMETRIC UNIT    4 - ATOMS IN THE UNIT CELL:   32
     ATOM                 X/A                 Y/B                 Z/C
 *******************************************************************************
      1 T   6 C     5.911898447340E-02  5.750517513864E-02 -8.226762271938E-02
      2 F   6 C    -5.911898447340E-02  4.424948248614E-01  4.177323772806E-01
      3 F   6 C    -4.408810155266E-01 -5.750517513864E-02 -4.177323772806E-01
      4 F   6 C     4.408810155266E-01 -4.424948248614E-01  8.226762271938E-02
      5 F   6 C    -5.911898447340E-02 -5.750517513864E-02  8.226762271938E-02
      6 F   6 C     5.911898447340E-02 -4.424948248614E-01 -4.177323772806E-01
      7 F   6 C     4.408810155266E-01  5.750517513864E-02  4.177323772806E-01
      8 F   6 C    -4.408810155266E-01  4.424948248614E-01 -8.226762271938E-02
      9 T   1 H     9.845800792887E-02  3.016713662044E-01 -1.470361735677E-01
     10 F   1 H    -9.845800792887E-02  1.983286337956E-01  3.529638264323E-01
     11 F   1 H    -4.015419920711E-01 -3.016713662044E-01 -3.529638264323E-01
     12 F   1 H     4.015419920711E-01 -1.983286337956E-01  1.470361735677E-01
     13 F   1 H    -9.845800792887E-02 -3.016713662044E-01  1.470361735677E-01
     14 F   1 H     9.845800792887E-02 -1.983286337956E-01 -3.529638264323E-01
     15 F   1 H     4.015419920711E-01  3.016713662044E-01  3.529638264323E-01
     16 F   1 H    -4.015419920711E-01  1.983286337956E-01 -1.470361735677E-01
     17 T   8 O     1.602337222665E-01 -4.139893216124E-03 -2.297037633723E-01
     18 F   8 O    -1.602337222665E-01 -4.958601067839E-01  2.702962366277E-01
     19 F   8 O    -3.397662777335E-01  4.139893216124E-03 -2.702962366277E-01
     20 F   8 O     3.397662777335E-01  4.958601067839E-01  2.297037633723E-01
     21 F   8 O    -1.602337222665E-01  4.139893216124E-03  2.297037633723E-01
     22 F   8 O     1.602337222665E-01  4.958601067839E-01 -2.702962366277E-01
     23 F   8 O     3.397662777335E-01 -4.139893216124E-03  2.702962366277E-01
     24 F   8 O    -3.397662777335E-01 -4.958601067839E-01 -2.297037633723E-01
     25 T   8 O     3.258414661148E-02  2.222176665805E-01 -3.975986057474E-02
     26 F   8 O    -3.258414661148E-02  2.777823334195E-01  4.602401394253E-01
     27 F   8 O    -4.674158533885E-01 -2.222176665805E-01 -4.602401394253E-01
     28 F   8 O     4.674158533885E-01 -2.777823334195E-01  3.975986057474E-02
     29 F   8 O    -3.258414661148E-02 -2.222176665805E-01  3.975986057474E-02
     30 F   8 O     3.258414661148E-02 -2.777823334195E-01 -4.602401394253E-01
     31 F   8 O     4.674158533885E-01  2.222176665805E-01  4.602401394253E-01
     32 F   8 O    -4.674158533885E-01  2.777823334195E-01 -3.975986057474E-02
```

--- crystal - beta

```
*******************************************************************************
 LATTICE PARAMETERS (ANGSTROMS AND DEGREES) - BOHR = 0.5291772083 ANGSTROM
 PRIMITIVE CELL - CENTRING CODE 1/0 VOLUME=   151.260708 - DENSITY  1.976 g/cm^3
         A              B              C           ALPHA      BETA       GAMMA
     5.34399446     5.99176883     5.24879674    90.000000 115.841166  90.000000
 *******************************************************************************
 ATOMS IN THE ASYMMETRIC UNIT    4 - ATOMS IN THE UNIT CELL:   16
     ATOM                 X/A                 Y/B                 Z/C
 *******************************************************************************
      1 T   6 C     1.443403379399E-01  2.044620008454E-03  9.216389088675E-04
      2 F   6 C    -1.443403379399E-01 -4.979553799915E-01  4.990783610911E-01
      3 F   6 C    -1.443403379399E-01 -2.044620008454E-03 -9.216389088675E-04
      4 F   6 C     1.443403379399E-01  4.979553799915E-01 -4.990783610911E-01
      5 T   1 H    -4.863444555594E-01 -1.013978805956E-01  2.085115389719E-01
      6 F   1 H     4.863444555594E-01  3.986021194044E-01  2.914884610281E-01
      7 F   1 H     4.863444555594E-01  1.013978805956E-01 -2.085115389719E-01
      8 F   1 H    -4.863444555594E-01 -3.986021194044E-01 -2.914884610281E-01
      9 T   8 O     1.856305486298E-01  1.016263809227E-01 -1.792550932739E-01
     10 F   8 O    -1.856305486298E-01 -3.983736190773E-01 -3.207449067261E-01
     11 F   8 O    -1.856305486298E-01 -1.016263809227E-01  1.792550932739E-01
     12 F   8 O     1.856305486298E-01  3.983736190773E-01  3.207449067261E-01
     13 T   8 O     3.294524310647E-01 -1.074968128959E-01  2.146080410523E-01
     14 F   8 O    -3.294524310647E-01  3.925031871041E-01  2.853919589477E-01
     15 F   8 O    -3.294524310647E-01  1.074968128959E-01 -2.146080410523E-01
     16 F   8 O     3.294524310647E-01 -3.925031871041E-01 -2.853919589477E-01
```

--- molecule

```
      1 T   6 C     3.777231540940E-01  4.397070023033E-01 -5.127588204347E-01
      2 T   8 O     1.040412800332E+00  1.229492998058E-02 -1.415078288462E+00
      3 T   8 O     1.810104186820E-01  1.737107154109E+00 -2.423155495603E-01
      4 T   6 C    -3.777231540940E-01 -4.397070023033E-01  5.127588204347E-01
      5 T   1 H     6.694483232148E-01  2.249553666153E+00 -9.064190111345E-01
      6 T   8 O    -1.040412800332E+00 -1.229492998058E-02  1.415078288462E+00
      7 T   8 O    -1.810104186820E-01 -1.737107154109E+00  2.423155495603E-01
      8 T   1 H    -6.694483232148E-01 -2.249553666153E+00  9.064190111345E-01
```

******

Cyanamide
******

--- crystal

```
*******************************************************************************
 LATTICE PARAMETERS (ANGSTROMS AND DEGREES) - BOHR = 0.5291772083 ANGSTROM
 PRIMITIVE CELL - CENTRING CODE 1/0 VOLUME=   408.849651 - DENSITY  1.365 g/cm^3
         A              B              C           ALPHA      BETA       GAMMA
     6.74452341     6.66624545     9.09349986    90.000000  90.000000  90.000000
 *******************************************************************************
 ATOMS IN THE ASYMMETRIC UNIT    5 - ATOMS IN THE UNIT CELL:   40
     ATOM                 X/A                 Y/B                 Z/C
 *******************************************************************************

      1 T   7 N     1.433161553458E-01  1.678754660181E-01  1.047211978077E-01
      2 F   7 N     3.566838446542E-01 -1.678754660181E-01 -3.952788021923E-01
      3 F   7 N    -3.566838446542E-01  3.321245339819E-01 -1.047211978077E-01
      4 F   7 N    -1.433161553458E-01 -3.321245339819E-01  3.952788021923E-01
      5 F   7 N    -1.433161553458E-01 -1.678754660181E-01 -1.047211978077E-01
      6 F   7 N    -3.566838446542E-01  1.678754660181E-01  3.952788021923E-01
      7 F   7 N     3.566838446542E-01 -3.321245339819E-01  1.047211978077E-01
      8 F   7 N     1.433161553458E-01  3.321245339819E-01 -3.952788021923E-01
      9 T   7 N     1.475896317159E-01  8.805753515943E-04  3.465673389782E-01
     10 F   7 N     3.524103682841E-01 -8.805753515943E-04 -1.534326610218E-01
     11 F   7 N    -3.524103682841E-01  4.991194246484E-01 -3.465673389782E-01
     12 F   7 N    -1.475896317159E-01 -4.991194246484E-01  1.534326610218E-01
     13 F   7 N    -1.475896317159E-01 -8.805753515943E-04 -3.465673389782E-01
     14 F   7 N    -3.524103682841E-01  8.805753515943E-04  1.534326610218E-01
     15 F   7 N     3.524103682841E-01 -4.991194246484E-01  3.465673389782E-01
     16 F   7 N     1.475896317159E-01  4.991194246484E-01 -1.534326610218E-01
     17 T   6 C     1.448610784952E-01  7.776976020511E-02  2.318303470795E-01
     18 F   6 C     3.551389215048E-01 -7.776976020511E-02 -2.681696529205E-01
     19 F   6 C    -3.551389215048E-01  4.222302397949E-01 -2.318303470795E-01
     20 F   6 C    -1.448610784952E-01 -4.222302397949E-01  2.681696529205E-01
     21 F   6 C    -1.448610784952E-01 -7.776976020511E-02 -2.318303470795E-01
     22 F   6 C    -3.551389215048E-01  7.776976020511E-02  2.681696529205E-01
     23 F   6 C     3.551389215048E-01 -4.222302397949E-01  2.318303470795E-01
     24 F   6 C     1.448610784952E-01  4.222302397949E-01 -2.681696529205E-01
     25 T   1 H     2.088224222083E-01  1.024527293306E-01  1.533387443846E-02
     26 F   1 H     2.911775777917E-01 -1.024527293306E-01 -4.846661255615E-01
     27 F   1 H    -2.911775777917E-01  3.975472706694E-01 -1.533387443846E-02
     28 F   1 H    -2.088224222083E-01 -3.975472706694E-01  4.846661255615E-01
     29 F   1 H    -2.088224222083E-01 -1.024527293306E-01 -1.533387443846E-02
```

```
   30 F   1 H    -2.911775777917E-01  1.024527293306E-01  4.846661255615E-01
   31 F   1 H     2.911775777917E-01 -3.975472706694E-01  1.533387443846E-02
   32 F   1 H     2.088224222083E-01  3.975472706694E-01 -4.846661255615E-01
   33 T   1 H     4.673962208426E-02  2.839682185621E-01  9.105936688600E-02
   34 F   1 H     4.532603779157E-01 -2.839682185621E-01 -4.089406331140E-01
   35 F   1 H    -4.532603779157E-01  2.160317814379E-01 -9.105936688600E-02
   36 F   1 H    -4.673962208426E-02 -2.160317814379E-01  4.089406331140E-01
   37 F   1 H    -4.673962208426E-02 -2.839682185621E-01 -9.105936688600E-02
   38 F   1 H    -4.532603779157E-01  2.839682185621E-01  4.089406331140E-01
   39 F   1 H     4.532603779157E-01 -2.160317814379E-01  9.105936688600E-02
   40 F   1 H     4.673962208426E-02  2.160317814379E-01 -4.089406331140E-01


--- molecule

    1 T   7 N     1.048903808172E+00  1.197840701091E+00  9.666877438148E-01
    2 T   1 H     2.973465905923E-01  1.847623545396E+00  7.877784631066E-01
    3 T   1 H     1.345418997070E+00  6.693850170557E-01  1.592969592122E-01
    4 T   6 C     9.918003835204E-01  5.289656285067E-01  2.125161201450E+00
    5 T   7 N     9.792142371343E-01 -2.443650331930E-02  3.140504032299E+00

******
1,4-Cyclohexadione
******

--- crystal


 *******************************************************************************
 LATTICE PARAMETERS (ANGSTROMS AND DEGREES) - BOHR = 0.5291772083 ANGSTROM
 PRIMITIVE CELL - CENTRING CODE 1/0 VOLUME=   270.447644 - DENSITY  1.376 g/cm^3
         A              B              C           ALPHA      BETA       GAMMA
     6.62767157     6.13544714     6.73542230    90.000000  99.090129  90.000000
 *******************************************************************************
 ATOMS IN THE ASYMMETRIC UNIT   16 - ATOMS IN THE UNIT CELL:   32
     ATOM                 X/A                 Y/B                 Z/C
 *******************************************************************************
    1 T   6 C     4.306487718072E-01  1.749199146965E-01  4.492740886922E-01
    2 F   6 C    -4.306487718072E-01 -3.250800853035E-01 -4.492740886922E-01
    3 T   6 C    -4.831212287881E-01  1.775551043147E-01  2.542073550827E-01
    4 F   6 C     4.831212287881E-01 -3.224448956853E-01 -2.542073550827E-01
    5 T   6 C     3.568418107006E-01  2.191525526065E-01  6.777506584503E-02
    6 F   6 C    -3.568418107006E-01 -2.808474473935E-01 -6.777506584503E-02
    7 T   6 C     1.712997167217E-01  7.437182114388E-02  5.949419225348E-02
```

```
  8 F   6 C    -1.712997167217E-01 -4.256281788561E-01 -5.949419225348E-02
  9 T   6 C     1.005257604134E-01  2.715688348155E-02  2.582447048902E-01
 10 F   6 C    -1.005257604134E-01 -4.728431165185E-01 -2.582447048902E-01
 11 T   6 C     2.010893670113E-01  1.720625025940E-01  4.319391375339E-01
 12 F   6 C    -2.010893670113E-01 -3.279374974060E-01 -4.319391375339E-01
 13 T   1 H    -3.607953464721E-01  2.977762400636E-01  2.658153960877E-01
 14 F   1 H     3.607953464721E-01 -2.022237599364E-01 -2.658153960877E-01
 15 T   1 H    -4.126632971932E-01  1.799066841028E-02  2.440807302801E-01
 16 F   1 H     4.126632971932E-01 -4.820093315897E-01 -2.440807302801E-01
 17 T   1 H     2.997773401099E-01  3.861781969414E-01  7.359277673388E-02
 18 F   1 H    -2.997773401099E-01 -1.138218030586E-01 -7.359277673388E-02
 19 T   1 H     4.207853750976E-01  2.003930905777E-01 -7.056956318730E-02
 20 F   1 H    -4.207853750976E-01 -2.996069094223E-01  7.056956318730E-02
 21 T   1 H     1.363563037955E-01 -1.441548968524E-01  2.918757485568E-01
 22 F   1 H    -1.363563037955E-01  3.558451031476E-01 -2.918757485568E-01
 23 T   1 H    -6.495467881241E-02  4.309883541619E-02  2.369637391686E-01
 24 F   1 H     6.495467881241E-02 -4.569011645838E-01 -2.369637391686E-01
 25 T   1 H     1.511866575112E-01  3.409550104444E-01  4.025082192309E-01
 26 F   1 H    -1.511866575112E-01 -1.590449895556E-01 -4.025082192309E-01
 27 T   1 H     1.586180936621E-01  1.231711335362E-01 -4.247431439189E-01
 28 F   1 H    -1.586180936621E-01 -3.768288664638E-01  4.247431439189E-01
 29 T   8 O    -4.566634480463E-01  1.739562499879E-01 -3.889066965331E-01
 30 F   8 O     4.566634480463E-01 -3.260437500121E-01  3.889066965331E-01
 31 T   8 O     8.407413981622E-02 -2.983307362510E-03 -9.756443532230E-02
 32 F   8 O    -8.407413981622E-02  4.970166926375E-01  9.756443532230E-02
```

```
--- molecule

  1 T   6 C     2.362570224017E+00  1.194354301653E+00  2.997414273080E+00
  2 T   8 O     2.901103299168E+00  1.450001560881E+00  4.049400858128E+00
  3 T   6 C     8.523722364967E-01  1.025982538358E+00  2.878049470492E+00
  4 T   6 C     3.149605282853E+00  1.019068272724E+00  1.703881437286E+00
  5 T   1 H     4.679028141794E-01  6.673791449268E-01  3.833396559758E+00
  6 T   1 H     4.361482154985E-01  2.031177687677E+00  2.735401258749E+00
  7 T   6 C     4.352603121719E-01  1.189921067932E-01  1.711388912789E+00
  8 T   1 H     4.054163400265E+00  1.624926299144E+00  1.764820624939E+00
  9 T   1 H     3.479738110630E+00 -2.703529421575E-02  1.678503250592E+00
 10 T   6 C     2.326310899951E+00  1.336167941694E+00  4.471377720617E-01
 11 T   1 H    -6.485017270448E-01  5.340359656662E-02  1.610708841095E+00
 12 T   1 H     7.913558407778E-01 -9.035238972120E-01  1.889240827282E+00
 13 T   6 C     1.019467017948E+00  5.551498743820E-01  3.727855997920E-01
 14 T   1 H     2.890064314265E+00  1.154962538012E+00 -4.685062341351E-01
 15 T   1 H     2.055781276148E+00  2.399549642951E+00  4.379456348095E-01
```

```
    16 T   8 O     4.815523590601E-01  2.980798802883E-01 -6.791789027681E-01
```

```
******
Imidazole
******

--- crystal
```

```
 *******************************************************************************
 LATTICE PARAMETERS (ANGSTROMS AND DEGREES) - BOHR = 0.5291772083 ANGSTROM
 PRIMITIVE CELL - CENTRING CODE 1/0 VOLUME=   334.604032 - DENSITY  1.351 g/cm^3
         A              B              C           ALPHA      BETA       GAMMA
     7.46243274     5.28792960     9.78600881    90.000000 119.947653  90.000000
 *******************************************************************************
 ATOMS IN THE ASYMMETRIC UNIT    9 - ATOMS IN THE UNIT CELL:   36
     ATOM                 X/A                 Y/B                 Z/C
 *******************************************************************************
      1 T   7 N     2.190972946595E-01  3.375088791229E-01  8.968267368658E-02
      2 F   7 N    -2.190972946595E-01 -1.624911208771E-01  4.103173263134E-01
      3 F   7 N    -2.190972946595E-01 -3.375088791229E-01 -8.968267368658E-02
      4 F   7 N     2.190972946595E-01  1.624911208771E-01 -4.103173263134E-01
      5 T   6 C     1.581194849459E-01  2.190502323648E-01  1.817323409265E-01
      6 F   6 C    -1.581194849459E-01 -2.809497676352E-01  3.182676590735E-01
      7 F   6 C    -1.581194849459E-01 -2.190502323648E-01 -1.817323409265E-01
      8 F   6 C     1.581194849459E-01  2.809497676352E-01 -3.182676590735E-01
      9 T   7 N     2.094043933689E-01  3.495623399898E-01  3.115659853181E-01
     10 F   7 N    -2.094043933689E-01 -1.504376600102E-01  1.884340146819E-01
     11 F   7 N    -2.094043933689E-01 -3.495623399898E-01 -3.115659853181E-01
     12 F   7 N     2.094043933689E-01  1.504376600102E-01 -1.884340146819E-01
     13 T   6 C     3.084593421935E-01 -4.374921342851E-01  3.016021950090E-01
     14 F   6 C    -3.084593421935E-01  6.250786571490E-02  1.983978049910E-01
     15 F   6 C    -3.084593421935E-01  4.374921342851E-01 -3.016021950090E-01
     16 F   6 C     3.084593421935E-01 -6.250786571490E-02 -1.983978049910E-01
     17 T   6 C     3.156318909171E-01 -4.426450803781E-01  1.648145466515E-01
     18 F   6 C    -3.156318909171E-01  5.735491962190E-02  3.351854533485E-01
     19 F   6 C    -3.156318909171E-01  4.426450803781E-01 -1.648145466515E-01
     20 F   6 C     3.156318909171E-01 -5.735491962190E-02 -3.351854533485E-01
     21 T   1 H     2.048185547029E-01  2.701745779443E-01 -1.514226125498E-02
     22 F   1 H    -2.048185547029E-01 -2.298254220557E-01 -4.848577387450E-01
     23 F   1 H    -2.048185547029E-01 -2.701745779443E-01  1.514226125498E-02
     24 F   1 H     2.048185547029E-01  2.298254220557E-01  4.848577387450E-01
     25 T   1 H     7.919948652555E-02  4.004666374045E-02  1.531558695631E-01
```

```
    26 F   1 H    -7.919948652555E-02 -4.599533362596E-01  3.468441304369E-01
    27 F   1 H    -7.919948652555E-02 -4.004666374045E-02 -1.531558695631E-01
    28 F   1 H     7.919948652555E-02  4.599533362596E-01 -3.468441304369E-01
    29 T   1 H     3.686845183630E-01 -2.953964142436E-01  3.922041281840E-01
    30 F   1 H    -3.686845183630E-01  2.046035857564E-01  1.077958718160E-01
    31 F   1 H    -3.686845183630E-01  2.953964142436E-01 -3.922041281840E-01
    32 F   1 H     3.686845183630E-01 -2.046035857564E-01 -1.077958718160E-01
    33 T   1 H     3.803651777467E-01 -3.118485275949E-01  1.171541739593E-01
    34 F   1 H    -3.803651777467E-01  1.881514724051E-01  3.828458260407E-01
    35 F   1 H    -3.803651777467E-01  3.118485275949E-01 -1.171541739593E-01
    36 F   1 H     3.803651777467E-01 -1.881514724051E-01 -3.828458260407E-01


--- molecule

     1 T   7 N     1.212971179749E+00  1.779184192815E+00  7.646400273121E-01
     2 T   1 H     1.568692773390E+00  1.447892693618E+00 -1.171229294398E-01
     3 T   6 C     2.911949794395E-01  1.154314897746E+00  1.555474364994E+00
     4 T   6 C     1.565596796768E+00  2.951581934422E+00  1.398462046797E+00
     5 T   1 H    -1.519670560659E-01  2.073419891563E-01  1.291604177951E+00
     6 T   7 N     3.791245831254E-02  1.846735216270E+00  2.639247452408E+00
     7 T   1 H     2.283471273597E+00  3.637561074995E+00  9.835772589693E-01
     8 T   6 C     8.284157280965E-01  2.969823655741E+00  2.551064868359E+00
     9 T   1 H     8.186103224772E-01  3.724045144276E+00  3.320639753758E+00

******
Acetic Acid
******

--- crystal


 *******************************************************************************
 LATTICE PARAMETERS (ANGSTROMS AND DEGREES) - BOHR = 0.5291772083 ANGSTROM
 PRIMITIVE CELL - CENTRING CODE 1/0 VOLUME=   288.746514 - DENSITY  1.381 g/cm^3
         A              B              C           ALPHA      BETA       GAMMA
    13.21315981     3.84238784     5.68733568    90.000000  90.000000  90.000000
 *******************************************************************************
 ATOMS IN THE ASYMMETRIC UNIT    8 - ATOMS IN THE UNIT CELL:   32
     ATOM                 X/A                 Y/B                 Z/C
 *******************************************************************************
     1 T   8 O     3.751906657218E-01 -1.116127452849E-01  2.921403525752E-03
     2 F   8 O    -3.751906657218E-01  1.116127452849E-01 -4.970785964742E-01
     3 F   8 O     1.248093342782E-01  3.883872547151E-01 -4.970785964742E-01
```

```
     4 F   8 O    -1.248093342782E-01 -3.883872547151E-01  2.921403525752E-03
     5 T   8 O     2.478107184188E-01 -3.991549494859E-01  1.693859095692E-01
     6 F   8 O    -2.478107184188E-01  3.991549494859E-01 -3.306140904308E-01
     7 F   8 O     2.521892815812E-01  1.008450505141E-01 -3.306140904308E-01
     8 F   8 O    -2.521892815812E-01 -1.008450505141E-01  1.693859095692E-01
     9 T   1 H     3.223726324347E-01 -5.121605306776E-02 -1.168386774894E-01
    10 F   1 H    -3.223726324347E-01  5.121605306776E-02  3.831613225106E-01
    11 F   1 H     1.776273675653E-01  4.487839469322E-01  3.831613225106E-01
    12 F   1 H    -1.776273675653E-01 -4.487839469322E-01 -1.168386774894E-01
    13 T   1 H     3.721487844072E-01  4.750636742237E-01 -4.938320622603E-01
    14 F   1 H    -3.721487844072E-01 -4.750636742237E-01  6.167937739700E-03
    15 F   1 H     1.278512155928E-01 -2.493632577630E-02  6.167937739700E-03
    16 F   1 H    -1.278512155928E-01  2.493632577630E-02 -4.938320622603E-01
    17 T   1 H     4.687307234846E-01  4.389099611609E-01  2.908135830398E-01
    18 F   1 H    -4.687307234846E-01 -4.389099611609E-01 -2.091864169602E-01
    19 F   1 H     3.126927651540E-02 -6.109003883910E-02 -2.091864169602E-01
    20 F   1 H    -3.126927651540E-02  6.109003883910E-02  2.908135830398E-01
    21 T   1 H     4.479139488925E-01 -1.578770927648E-01  4.262483309267E-01
    22 F   1 H    -4.479139488925E-01  1.578770927648E-01 -7.375166907330E-02
    23 F   1 H     5.208605110750E-02  3.421229072352E-01 -7.375166907330E-02
    24 F   1 H    -5.208605110750E-02 -3.421229072352E-01  4.262483309267E-01
    25 T   6 C     3.364064124016E-01 -3.035035917791E-01  1.735935435380E-01
    26 F   6 C    -3.364064124016E-01  3.035035917791E-01 -3.264064564620E-01
    27 F   6 C     1.635935875984E-01  1.964964082209E-01 -3.264064564620E-01
    28 F   6 C    -1.635935875984E-01 -1.964964082209E-01  1.735935435380E-01
    29 T   6 C     4.103407329385E-01 -3.919333855850E-01  3.633079691501E-01
    30 F   6 C    -4.103407329385E-01  3.919333855850E-01 -1.366920308499E-01
    31 F   6 C     8.965926706150E-02  1.080666144150E-01 -1.366920308499E-01
    32 F   6 C    -8.965926706150E-02 -1.080666144150E-01  3.633079691501E-01

--- molecule

     1 T   8 O     4.983368673976E+00 -4.473884597889E-01 -1.214610331341E-02
     2 T   1 H     4.282918874246E+00 -2.586098863110E-01 -6.563718656122E-01
     3 T   6 C     4.416178494901E+00 -1.160500153495E+00  9.967011322867E-01
     4 T   8 O     3.248526841090E+00 -1.454660235061E+00  1.000590370124E+00
     5 T   6 C     5.423018321011E+00 -1.511251489035E+00  2.058152975033E+00
     6 T   1 H     4.934964157811E+00 -2.071083017849E+00  2.851374398898E+00
     7 T   1 H     5.869547198438E+00 -6.016213155233E-01  2.463624534179E+00
     8 T   1 H     6.228778675372E+00 -2.105943065991E+00  1.624001219012E+00

******
Succinic Acid
******
```

--- crystal

```
 *******************************************************************************
 LATTICE PARAMETERS (ANGSTROMS AND DEGREES) - BOHR = 0.5291772083 ANGSTROM
 PRIMITIVE CELL - CENTRING CODE 1/0 VOLUME=   233.904416 - DENSITY  1.676 g/cm^3
         A              B              C           ALPHA      BETA       GAMMA
     5.47129300     8.53402094     5.01925899    90.000000  93.572707  90.000000
 *******************************************************************************
 ATOMS IN THE ASYMMETRIC UNIT    7 - ATOMS IN THE UNIT CELL:   28
     ATOM                 X/A                 Y/B                 Z/C
 *******************************************************************************
      1 T   8 O    -2.540396342707E-01  4.242090599159E-01  1.106793506367E-01
      2 F   8 O     2.540396342707E-01 -7.579094008410E-02  3.893206493633E-01
      3 F   8 O     2.540396342707E-01 -4.242090599159E-01 -1.106793506367E-01
      4 F   8 O    -2.540396342707E-01  7.579094008410E-02 -3.893206493633E-01
      5 T   8 O    -6.334770079952E-02 -3.578874641943E-01  2.620628333417E-01
      6 F   8 O     6.334770079952E-02  1.421125358057E-01  2.379371666583E-01
      7 F   8 O     6.334770079952E-02  3.578874641943E-01 -2.620628333417E-01
      8 F   8 O    -6.334770079952E-02 -1.421125358057E-01 -2.379371666583E-01
      9 T   6 C    -2.387546608205E-01 -4.644952333099E-01  2.648741963378E-01
     10 F   6 C     2.387546608205E-01  3.550476669010E-02  2.351258036622E-01
     11 F   6 C     2.387546608205E-01  4.644952333099E-01 -2.648741963378E-01
     12 F   6 C    -2.387546608205E-01 -3.550476669010E-02 -2.351258036622E-01
     13 T   6 C     4.159536080015E-01  4.315031566123E-01 -4.749026268643E-01
     14 F   6 C    -4.159536080015E-01 -6.849684338770E-02 -2.509737313570E-02
     15 F   6 C    -4.159536080015E-01 -4.315031566123E-01  4.749026268643E-01
     16 F   6 C     4.159536080015E-01  6.849684338770E-02  2.509737313570E-02
     17 T   1 H    -3.102403582365E-01 -3.964539864122E-01 -3.440852597565E-01
     18 F   1 H     3.102403582365E-01  1.035460135878E-01 -1.559147402435E-01
     19 F   1 H     3.102403582365E-01  3.964539864122E-01  3.440852597565E-01
     20 F   1 H    -3.102403582365E-01 -1.035460135878E-01  1.559147402435E-01
     21 T   1 H     4.787154117012E-01 -3.279225778127E-01  4.098004479814E-01
     22 F   1 H    -4.787154117012E-01  1.720774221873E-01  9.019955201860E-02
     23 F   1 H    -4.787154117012E-01  3.279225778127E-01 -4.098004479814E-01
     24 F   1 H     4.787154117012E-01 -1.720774221873E-01 -9.019955201860E-02
     25 T   1 H     4.758747473696E-02 -3.774840442781E-01  1.152628531038E-01
     26 F   1 H    -4.758747473696E-02  1.225159557219E-01  3.847371468962E-01
     27 F   1 H    -4.758747473696E-02  3.774840442781E-01 -1.152628531038E-01
     28 F   1 H     4.758747473696E-02 -1.225159557219E-01 -3.847371468962E-01
```

--- molecule

```
      1 T   8 O    -1.300643219016E+00  3.544730256550E+00  6.802475684690E-01
      2 T   6 C    -1.364498628696E+00  4.542349479543E+00  1.352455212492E+00
      3 T   8 O    -4.839952474583E-01  5.562910478299E+00  1.227135614464E+00
      4 T   6 C    -2.393450648453E+00  4.835099738094E+00  2.415627412946E+00
      5 T   1 H     1.411850994036E-01  5.310278604595E+00  5.316690992560E-01
      6 T   1 H    -1.869625414119E+00  5.052179302169E+00  3.348330342641E+00
      7 T   1 H    -2.898918807230E+00  5.766291208242E+00  2.152193012009E+00
      8 T   6 C    -3.390524771210E+00  3.699105282082E+00  2.594069552565E+00
      9 T   1 H    -3.914060268623E+00  3.481899140051E+00  1.661251499697E+00
     10 T   1 H    -2.885111054132E+00  2.767995470331E+00  2.857753883612E+00
     11 T   6 C    -4.419902060299E+00  3.991850219195E+00  3.656801647604E+00
     12 T   8 O    -4.484728747730E+00  4.989927469384E+00  4.328294686213E+00
     13 T   8 O    -5.299248924041E+00  2.970402540382E+00  3.782870130933E+00
     14 T   1 H    -5.924957678503E+00  3.223127391083E+00  4.477829658652E+00
```

```
****
Cytosine
****

--- crystal

 *******************************************************************************
 LATTICE PARAMETERS (ANGSTROMS AND DEGREES) - BOHR = 0.5291772083 ANGSTROM
 PRIMITIVE CELL - CENTRING CODE 1/0 VOLUME=   451.393326 - DENSITY  1.634 g/cm^3
         A              B              C           ALPHA      BETA       GAMMA
    13.00717346     9.42684406     3.68133957    90.000000  90.000000  90.000000
 *******************************************************************************
 ATOMS IN THE ASYMMETRIC UNIT   13 - ATOMS IN THE UNIT CELL:   52
     ATOM                 X/A                 Y/B                 Z/C
 *******************************************************************************
      1 T   6 C    -1.710294989999E-02  1.533868523113E-01  3.352828504645E-01
      2 F   6 C    -4.828970501000E-01 -1.533868523113E-01 -1.647171495355E-01
      3 F   6 C     4.828970501000E-01  3.466131476887E-01 -3.352828504645E-01
      4 F   6 C     1.710294989999E-02 -3.466131476887E-01  1.647171495355E-01
      5 T   6 C     1.300616194741E-01  2.625900184175E-01 -4.315039873902E-01
      6 F   6 C     3.699383805259E-01 -2.625900184175E-01  6.849601260980E-02
      7 F   6 C    -3.699383805259E-01  2.374099815825E-01  4.315039873902E-01
      8 F   6 C    -1.300616194741E-01 -2.374099815825E-01 -6.849601260980E-02
      9 T   6 C     1.703241617419E-01  1.299539251940E-01 -3.096262007953E-01
     10 F   6 C     3.296758382581E-01 -1.299539251940E-01  1.903737992047E-01
     11 F   6 C    -3.296758382581E-01  3.700460748060E-01  3.096262007953E-01
     12 F   6 C    -1.703241617419E-01 -3.700460748060E-01 -1.903737992047E-01
```

```
13 T   6 C     1.124530350017E-01  1.345863058134E-02 -3.735412492377E-01
14 F   6 C     3.875469649983E-01 -1.345863058134E-02  1.264587507623E-01
15 F   6 C    -3.875469649983E-01  4.865413694187E-01  3.735412492377E-01
16 F   6 C    -1.124530350017E-01 -4.865413694187E-01 -1.264587507623E-01
17 T   1 H    -1.137632897035E-02 -6.827556664638E-02  3.526054453213E-01
18 F   1 H    -4.886236710296E-01  6.827556664638E-02 -1.473945546787E-01
19 F   1 H     4.886236710297E-01 -4.317244333536E-01 -3.526054453213E-01
20 F   1 H     1.137632897035E-02  4.317244333536E-01  1.473945546787E-01
21 T   1 H     1.544516504278E-01  4.762465153290E-01 -4.611494260378E-01
22 F   1 H     3.455483495722E-01 -4.762465153290E-01  3.885057396220E-02
23 F   1 H    -3.455483495722E-01  2.375348467100E-02  4.611494260378E-01
24 F   1 H    -1.544516504278E-01 -2.375348467100E-02 -3.885057396220E-02
25 T   1 H     2.581121897376E-01  3.750053317720E-01 -2.970266283693E-01
26 F   1 H     2.418878102624E-01 -3.750053317720E-01  2.029733716307E-01
27 F   1 H    -2.418878102624E-01  1.249946682280E-01  2.970266283693E-01
28 F   1 H    -2.581121897376E-01 -1.249946682280E-01 -2.029733716307E-01
29 T   1 H     2.428810800981E-01  1.247482549183E-01 -1.707597405321E-01
30 F   1 H     2.571189199019E-01 -1.247482549183E-01  3.292402594679E-01
31 F   1 H    -2.571189199019E-01  3.752517450817E-01  1.707597405321E-01
32 F   1 H    -2.428810800981E-01 -3.752517450817E-01 -3.292402594679E-01
33 T   1 H     1.337342489897E-01 -9.286520410182E-02 -2.930087053421E-01
34 F   1 H     3.662657510103E-01  9.286520410182E-02  2.069912946579E-01
35 F   1 H    -3.662657510103E-01 -4.071347958982E-01  2.930087053421E-01
36 F   1 H    -1.337342489897E-01  4.071347958982E-01 -2.069912946579E-01
37 T   7 N     2.122526401373E-02  2.429392061771E-02  4.527834374242E-01
38 F   7 N     4.787747359863E-01 -2.429392061771E-02 -4.721656257580E-02
39 F   7 N    -4.787747359863E-01  4.757060793823E-01 -4.527834374242E-01
40 F   7 N    -2.122526401373E-02 -4.757060793823E-01  4.721656257580E-02
41 T   7 N     3.878675260453E-02  2.717108891895E-01  4.019106402690E-01
42 F   7 N     4.612132473955E-01 -2.717108891895E-01 -9.808935973100E-02
43 F   7 N    -4.612132473955E-01  2.282891108105E-01 -4.019106402690E-01
44 F   7 N    -3.878675260453E-02 -2.282891108105E-01  9.808935973100E-02
45 T   7 N     1.835287439586E-01  3.816020752338E-01 -3.770847323402E-01
46 F   7 N     3.164712560414E-01 -3.816020752338E-01  1.229152676598E-01
47 F   7 N    -3.164712560414E-01  1.183979247662E-01  3.770847323402E-01
48 F   7 N    -1.835287439586E-01 -1.183979247662E-01 -1.229152676598E-01
49 T   8 O    -1.004688553394E-01  1.565654580989E-01  1.667620724699E-01
50 F   8 O    -3.995311446606E-01 -1.565654580989E-01 -3.332379275301E-01
51 F   8 O     3.995311446606E-01  3.434345419011E-01 -1.667620724699E-01
52 F   8 O     1.004688553394E-01 -3.434345419011E-01  3.332379275301E-01
```

```
--- molecule
```

```
      1 T   1 H     2.498950760440E-01  4.112623376324E+00  4.817657307555E-01
      2 T   1 H    -1.819536648545E+00  3.817666943019E+00 -6.382059536016E-01
      3 T   1 H    -3.200200331490E+00  5.857069375947E+00 -1.104244960479E+00
      4 T   1 H    -3.304441836486E+00  8.316061835171E+00 -7.673157473956E-01
      5 T   1 H    -1.998920581297E+00  9.172622397611E+00 -8.035872208804E-03
      6 T   6 C     2.213552291001E-01  6.169304963744E+00  7.299950762571E-01
      7 T   6 C    -1.508598561556E+00  4.812964936807E+00 -3.479173107336E-01
      8 T   6 C    -1.695216910274E+00  7.168397407790E+00 -1.452716934460E-01
      9 T   6 C    -2.250228360723E+00  5.918524166001E+00 -5.948551986493E-01
     10 T   7 N    -3.198478575149E-01  4.922812999219E+00  2.877371493539E-01
     11 T   7 N    -5.399943371939E-01  7.277642030593E+00  4.748978698160E-01
     12 T   7 N    -2.381387249876E+00  8.315881509877E+00 -3.730471390615E-01
     13 T   8 O     1.297622369811E+00  6.163628057896E+00  1.292798049393E+00
```

```
******
Pyrazole
******

--- crystal

 *******************************************************************************
 LATTICE PARAMETERS (ANGSTROMS AND DEGREES) - BOHR = 0.5291772083 ANGSTROM
 PRIMITIVE CELL - CENTRING CODE 1/0 VOLUME=   702.241875 - DENSITY  1.287 g/cm^3
         A              B              C           ALPHA      BETA       GAMMA
     8.05026337    12.82684891     6.80074757    90.000000  90.000000  90.000000
 *******************************************************************************
 ATOMS IN THE ASYMMETRIC UNIT   18 - ATOMS IN THE UNIT CELL:   72
     ATOM                 X/A                 Y/B                 Z/C
 *******************************************************************************
      1 T   6 C     2.989949493721E-01  5.781083282184E-02  3.889168609070E-01
      2 F   6 C    -2.010050506279E-01 -5.781083282184E-02 -3.889168609070E-01
      3 F   6 C     2.989949493721E-01  4.421891671782E-01 -1.110831390930E-01
      4 F   6 C    -2.010050506279E-01 -4.421891671782E-01  1.110831390930E-01
      5 T   6 C    -2.785505805943E-01  1.841428703726E-01 -7.784033585529E-02
      6 F   6 C     2.214494194057E-01 -1.841428703726E-01  7.784033585529E-02
      7 F   6 C    -2.785505805943E-01  3.158571296274E-01  4.221596641447E-01
      8 F   6 C     2.214494194057E-01 -3.158571296274E-01 -4.221596641447E-01
      9 T   6 C     4.049423488126E-01  1.156610362555E-01 -4.887059259027E-01
     10 F   6 C    -9.505765118740E-02 -1.156610362555E-01  4.887059259027E-01
     11 F   6 C     4.049423488126E-01  3.843389637445E-01  1.129407409730E-02
     12 F   6 C    -9.505765118740E-02 -3.843389637445E-01 -1.129407409730E-02
     13 T   6 C    -3.869501587751E-01  1.078354459353E-01 -4.446098369772E-03
```

14	F	6	C	1.130498412249E-01	-1.078354459353E-01	4.446098369772E-03
15	F	6	C	-3.869501587751E-01	3.921645540647E-01	4.955539016302E-01
16	F	6	C	1.130498412249E-01	-3.921645540647E-01	-4.955539016302E-01
17	T	6	C	3.080222335481E-01	1.969057157897E-01	-4.207517682595E-01
18	F	6	C	-1.919777664519E-01	-1.969057157897E-01	4.207517682595E-01
19	F	6	C	3.080222335481E-01	3.030942842103E-01	7.924823174050E-02
20	F	6	C	-1.919777664519E-01	-3.030942842103E-01	-7.924823174050E-02
21	T	6	C	-2.947776546750E-01	5.426974633292E-02	1.338184006669E-01
22	F	6	C	2.052223453250E-01	-5.426974633292E-02	-1.338184006669E-01
23	F	6	C	-2.947776546750E-01	4.457302536671E-01	-3.661815993331E-01
24	F	6	C	2.052223453250E-01	-4.457302536671E-01	3.661815993331E-01
25	T	1	H	5.390695794535E-02	2.337632430147E-01	-4.894107547391E-01
26	F	1	H	-4.460930420547E-01	-2.337632430147E-01	4.894107547391E-01
27	F	1	H	5.390695794535E-02	2.662367569853E-01	1.058924526090E-02
28	F	1	H	-4.460930420547E-01	-2.662367569853E-01	-1.058924526090E-02
29	T	1	H	-4.284478415413E-02	8.378233516401E-02	2.284872955468E-01
30	F	1	H	4.571552158459E-01	-8.378233516401E-02	-2.284872955468E-01
31	F	1	H	-4.284478415413E-02	4.162176648360E-01	-2.715127044532E-01
32	F	1	H	4.571552158459E-01	-4.162176648360E-01	2.715127044532E-01
33	T	1	H	3.370892878401E-01	2.612662910041E-01	-3.256073987426E-01
34	F	1	H	-1.629107121599E-01	-2.612662910041E-01	3.256073987426E-01
35	F	1	H	3.370892878401E-01	2.387337089959E-01	1.743926012574E-01
36	F	1	H	-1.629107121599E-01	-2.387337089959E-01	-1.743926012574E-01
37	T	1	H	-3.257014243585E-01	-1.041387798566E-02	2.275309880843E-01
38	F	1	H	1.742985756415E-01	1.041387798566E-02	-2.275309880843E-01
39	F	1	H	-3.257014243585E-01	-4.895861220143E-01	-2.724690119157E-01
40	F	1	H	1.742985756415E-01	4.895861220143E-01	2.724690119157E-01
41	T	1	H	-4.669784094409E-01	1.004705947930E-01	-4.553433552575E-01
42	F	1	H	3.302159055910E-02	-1.004705947930E-01	4.553433552575E-01
43	F	1	H	-4.669784094409E-01	3.995294052070E-01	4.465664474250E-02
44	F	1	H	3.302159055910E-02	-3.995294052070E-01	-4.465664474250E-02
45	T	1	H	4.862700582098E-01	9.401233086075E-02	-4.581828469010E-02
46	F	1	H	-1.372994179020E-02	-9.401233086075E-02	4.581828469010E-02
47	F	1	H	4.862700582098E-01	4.059876691393E-01	4.541817153099E-01
48	F	1	H	-1.372994179020E-02	-4.059876691393E-01	-4.541817153099E-01
49	T	1	H	3.248870126757E-01	-1.190870514826E-02	3.059705250543E-01
50	F	1	H	-1.751129873243E-01	1.190870514826E-02	-3.059705250543E-01
51	F	1	H	3.248870126757E-01	-4.880912948517E-01	-1.940294749457E-01
52	F	1	H	-1.751129873243E-01	4.880912948517E-01	1.940294749457E-01
53	T	1	H	-3.017740628382E-01	2.424869087496E-01	-1.880621219027E-01
54	F	1	H	1.982259371618E-01	-2.424869087496E-01	1.880621219027E-01
55	F	1	H	-3.017740628382E-01	2.575130912504E-01	3.119378780973E-01
56	F	1	H	1.982259371618E-01	-2.575130912504E-01	-3.119378780973E-01
57	T	7	N	1.556373399715E-01	1.847968496866E-01	-4.998093190909E-01

```
 58 F   7 N    -3.443626600285E-01 -1.847968496866E-01  4.998093190909E-01
 59 F   7 N     1.556373399715E-01  3.152031503134E-01  1.906809091001E-04
 60 F   7 N    -3.443626600285E-01 -3.152031503134E-01 -1.906809091001E-04
 61 T   7 N    -1.427175715150E-01  9.843173444357E-02  1.374170132409E-01
 62 F   7 N     3.572824284850E-01 -9.843173444357E-02 -1.374170132409E-01
 63 F   7 N    -1.427175715150E-01  4.015682655564E-01 -3.625829867591E-01
 64 F   7 N     3.572824284850E-01 -4.015682655564E-01  3.625829867591E-01
 65 T   7 N     1.477424399295E-01  1.002753687963E-01  3.822592788385E-01
 66 F   7 N    -3.522575600705E-01 -1.002753687963E-01 -3.822592788385E-01
 67 F   7 N     1.477424399295E-01  3.997246312037E-01 -1.177407211615E-01
 68 F   7 N    -3.522575600705E-01 -3.997246312037E-01  1.177407211615E-01
 69 T   7 N    -1.302379819536E-01  1.781672411248E-01  8.894589769756E-03
 70 F   7 N     3.697620180464E-01 -1.781672411248E-01 -8.894589769756E-03
 71 F   7 N    -1.302379819536E-01  3.218327588752E-01 -4.911054102302E-01
 72 F   7 N     3.697620180464E-01 -3.218327588752E-01  4.911054102302E-01


--- molecule

  1 T   6 C     2.393488384363E+00  7.487901238589E-01  2.644960208240E+00
  2 T   1 H     2.603071334569E+00 -1.512653380270E-01  2.089182853956E+00
  3 T   7 N     1.181641129643E+00  1.289144050427E+00  2.583575679658E+00
  4 T   6 C     3.258464456193E+00  1.489071998206E+00  3.479379974946E+00
  5 T   7 N     1.263267514293E+00  2.376230270568E+00  3.376790709988E+00
  6 T   1 H     4.289804810310E+00  1.287796791487E+00  3.713449488203E+00
  7 T   6 C     2.489342334452E+00  2.536535748483E+00  3.935389753249E+00
  8 T   1 H     4.501147957462E-01  2.958050162773E+00  3.489196821389E+00
  9 T   1 H     2.713646078680E+00  3.358640804387E+00  4.594485334100E+00
```
